# A molecular index for biological age identified from the metabolome and senescence‐associated secretome in humans

**DOI:** 10.1111/acel.14104

**Published:** 2024-03-07

**Authors:** Shruthi Hamsanathan, Tamil Anthonymuthu, Denise Prosser, Anna Lokshin, Susan L. Greenspan, Neil M. Resnick, Subashan Perera, Satoshi Okawa, Giri Narasimhan, Aditi U. Gurkar

**Affiliations:** ^1^ Aging Institute of UPMC and the University of Pittsburgh School of Medicine Pittsburgh Pennsylvania USA; ^2^ Department of Critical Care Medicine University of Pittsburgh School of Medicine Pittsburgh Pennsylvania USA; ^3^ Department of Medicine University of Pittsburgh Medical Center and University of Pittsburgh Cancer Institute Pittsburgh Pennsylvania USA; ^4^ Division of Geriatric Medicine, Department of Medicine University of Pittsburgh School of Medicine Pittsburgh Pennsylvania USA; ^5^ Department of Biostatistics University of Pittsburgh Graduate School of Public Health Pittsburgh Pennsylvania USA; ^6^ Pittsburgh Heart, Lung, and Blood Vascular Medicine Institute University of Pittsburgh School of Medicine Pittsburgh Pennsylvania USA; ^7^ Department of Computational and Systems Biology University of Pittsburgh School of Medicine Pittsburgh Pennsylvania USA; ^8^ McGowan Institute for Regenerative Medicine University of Pittsburgh School of Medicine Pittsburgh Pennsylvania USA; ^9^ Bioinformatics Research Group (BioRG), School of Computing and Information Sciences, Biomolecular Sciences Institute Florida International University Miami Florida USA

**Keywords:** aging, biological age, cellular senescence, metabolomics, SASP

## Abstract

Unlike chronological age, biological age is a strong indicator of health of an individual. However, the molecular fingerprint associated with biological age is ill‐defined. To define a high‐resolution signature of biological age, we analyzed metabolome, circulating senescence‐associated secretome (SASP)/inflammation markers and the interaction between them, from a cohort of healthy and rapid agers. The balance between two fatty acid oxidation mechanisms, β‐oxidation and ω‐oxidation, associated with the extent of functional aging. Furthermore, a panel of 25 metabolites, Healthy Aging Metabolic (HAM) index, predicted healthy agers regardless of gender and race. HAM index was also validated in an independent cohort. Causal inference with machine learning implied three metabolites, β‐cryptoxanthin, prolylhydroxyproline, and eicosenoylcarnitine as putative drivers of biological aging. Multiple SASP markers were also elevated in rapid agers. Together, our findings reveal that a network of metabolic pathways underlie biological aging, and the HAM index could serve as a predictor of phenotypic aging in humans.

## Graphical abstract

Due to unprecedented growth of the world's aging population, the number of individuals affected by chronic diseases and the resulting socio‐economic burden and disparities will continue to rise. Identification of molecular markers that reflect rapid biological aging and a diagnostic measure that can easily be applied in the clinic are critically needed. Here, we combined metabolome and the SASP to provide a high‐resolution view of such a complex phenotype.

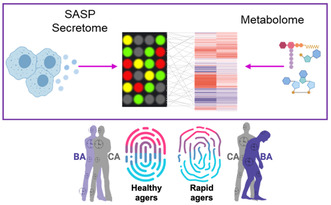

**Abbreviations**
AUC: area under the curve
BMI: body mass index
cAMP: cyclic adenosine monophosphate
CRP: C‐reactive protein
DCAs: dicarboxylic acids
HAM: healthy aging metabolic index
HIF1a: hypoxia‐inducible factor 1‐alpha
MCP1: monocyte chemoattractant protein 1
MMP‐1: matrix metalloproteinase
MOCA: montreal cognitive assessment
OPLS‐DA: orthogonal partial least square‐discriminant analysis
PAI‐1: plasminogen activator inhibitor‐1
RMSE: root mean square cross validation error
ROC: receiver operative characteristic
ROS: reactive oxygen species
SAM: S adenosyl methionine
SASP: senescence‐associated secretory phenotype
UPLC–MS/MS: ultrahigh‐performance liquid chromatography–tandem mass spectroscopy
VIP: variable importance of the projection
VLSFA: very long chain saturated fatty acids
WRAP: wisconsin registry of Alzheimer's patients

## 1 INTRODUCTION

Chronological age is the principal risk factor for several chronic diseases. However, at a population level, individuals do not exhibit phenotypic aging at the same rate (Lunenfeld & Stratton, 2013). Rapid agers display a faster rate of biological or phenotypic aging relative to their chronological age. Detecting a rapid ager early on will not only present intervention opportunities, but also reduce the socioeconomic burden on society. In a society with an ever‐increasing aging population, it is therefore critical to identify molecular markers that reflect rapid biological aging. However, it is challenging to differentiate biological age from chronological age. A number of studies have identified signals that measure aging in general, with an attempt to reflect biological age. Candidate markers for biological age measures include individual phenotypic parameters such as low‐grade inflammation, DNA methylation, muscle mass and strength, frailty, neuroendocrine function, and immune markers (Franceschi & Campisi, 2014; Gruenewald et al., 2006; Horvath et al., 2022; Peterson et al., 2016; Walston et al., 2006). While these markers are successful in depicting the individual events in the aging process, they do not truly represent the complexity of biological aging.

Biological aging is multifaceted, arising from complex genetic traits and influenced by epigenetics, environment, diet, and exercise. Metabolomics is a powerful tool that has the ability to capture the complete set of circulating metabolites and is particularly suited to account for biological age. One potential advantage of metabolomics over other “omic” approaches is that metabolites are the final downstream products, and changes are closely related to the immediate (patho)physiologic state of an individual. Metabolites inform on multiple biological processes, including cellular macromolecules (nucleic acids, amino acids, lipids, etc.), nutrients, drug, and intermediates. The dimensionality of metabolites in capturing both genetic and nongenetic features often influenced by disease, environment, epigenetics, microbiome, and lifestyle factors makes it an ideal candidate for measuring a complex process such as aging (Aguiar‐Pulido et al., 2016). Furthermore, a number of interventions that have shown promise and are directed to improve health span, such as calorie restriction, time‐restricted feeding, and intermittent fasting are known to target metabolic pathways (de Cabo & Mattson, 2019; Hatori et al., 2012; Redman et al., 2018). Metabolomic profiling is also advantageous over the widely studied DNA and protein‐based clocks because they provide information on metabolic pathways in addition to biomarkers predicting age‐related morbidity, which could be further explored to tailor interventions.

In addition to metabolomic alterations, changes in senescence‐associated inflammatory markers are another important but underexplored drivers of biological age. When cells encounter stochastic macromolecular damage or stress, they can enter a state of permanent cell cycle arrest known as senescence (Gurkar et al., 2023; Hamsanathan et al., 2019; Niedernhofer et al., 2018). It is evident that there is an increase in the number of senescent cells with age. These senescent cells primarily secrete multiple inflammatory cytokines and elicit low‐grade inflammation. This pro‐inflammation status leads to subsequent age‐related diseases and morbidity (He & Sharpless, 2017). Indeed, modulation of secretory pathways and clearance of senescent cells has emerged as an attractive therapeutic strategy for aging (Childs et al., 2015; Zhu et al., 2015). However, translation to human applications is impeded by the fact that we do not know how prevalent senescent cells are in vivo and whether these cells promote biological aging in humans. The SASP secretome is considered to be a detrimental hallmark of senescent cells and leveraging such circulating SASP and inflammatory factors could potentially inform about biological age (Fielding et al., 2022). Currently, none of the available markers are sufficient on their own for conclusively fingerprinting a complex process such as biological age.

On the analytical end, correlations, even significant ones, are relatively easy to detect, but causal relationships are not. In a complex multifactorial physiological process like aging, it is challenging to detect what might be causative of healthy or rapid biological aging. Causal inference with machine learning is a tool that uses the concept of conditional statistical independence to computationally filter out many correlational relationships that are not causal (Glymour et al., 2019; Lecca, 2021; Pearl, 2010). Such causal inferencing is of great interest, especially applied to biological age, as it will not only help understand the mechanisms underlying the process, but also help unravel how dynamically biological age responds to potential interventions. While there is some evidence for metabolic changes and inflammation with chronological age, do these changes contribute and are causal to either rapid biological aging or healthy aging is unclear. Identifying the unique molecular markers responsible for rapid biological aging would be crucial for understanding the biopathophysiology of the aging process and to guide appropriate interventional strategies. We adopted an integrated approach in this study, and simultaneously measured SASP/inflammatory factors and metabolites from serum samples to generate a fingerprint for biological aging.

## 2 RESULTS

### 2.1 Comprehensive metabolomic assessment to assess biological aging

**FIGURE 1:**
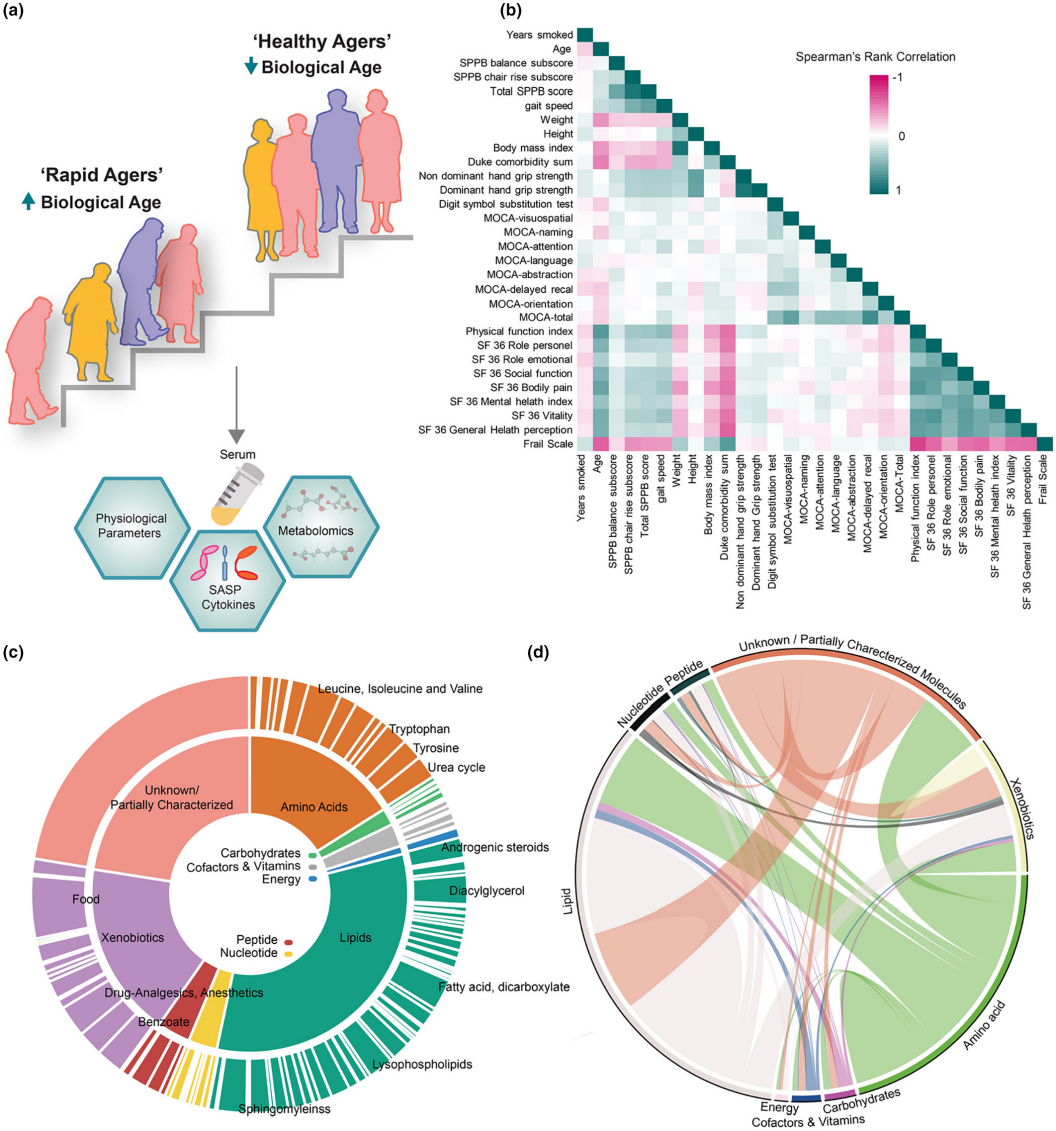
Global metabolic profiling of SOLVE‐IT study cohort. (a) Study design: A total of 196 participants were recruited through the Claude D. Pepper Older Americans Independence Center at University of Pittsburgh. All study participants were medically‐stable volunteers who were independently mobile. Both groups were controlled for demographic and clinical features. Using their performance‐based mobility measures, the participants were grouped as “rapid” or “healthy” agers. Serum metabolite profiling was performed using high‐resolution LC‐MS/MS‐based untargeted metabolomics analysis. SASP was measured by Luminex High Performance Assay and proinflammatory markers were measured as previously described (Langmann et al., 2017). The data from clinical features of phenotypic aging, SASP, cytokines, and metabolites were integrated using statistical approaches to generate a molecular index for Biological Aging. (b) Heat map shows the Spearman's rank correlations coefficients (*ρ* or rs') among the demographic and clinical parameters of the SOLVE‐IT cohort. Teal indicates positive and pink indicates negative correlation associations. MOCA, Montreal Cognitive Assessment; SF‐36, Short Form 36 (c) Stacked donut chart showing the distribution of metabolites in super (inner donut) and sub metabolic (outer donut) pathways as identified in KEGG (refer Table S3). (d) Number of connections deemed important (FDR corrected *p*‐value for *ρ* <0.2) between the metabolites is highlighted in the chord diagram. The thickness of the chord is proportional to the number of relationships.

Biological age, not chronological age, captures one's physical and functional ability and is a determinant of health span (years lived in good health). We took an integrated approach encompassing high‐resolution metabolomics combined with a panel of SASP and proinflammatory markers in the serum, to define a molecular index for biological aging. Walking ability and gait speed have been previously associated with predictors of survival, cognitive function, and general health in the elderly (Cooper et al., 2014; Kikkert et al., 2016; Studenski et al., 2011). Indeed, walking ability reflects an integrated assessment of cardiovascular fitness, muscle strength, neurological and joint function and is currently the single best predictor in humans for hospitalization, functional decline, disability, surgical complications, institutionalization, and death (Afilalo et al., 2016; Guralnik et al., 1995; Perera et al., 2016; Studenski et al., 2011). The “SOLVE‐IT” cohort consisted of 196 total participants, 98 individuals above 75 years old that showed good walking ability (walk up a flight of stairs and walk for 15 min without resting). The individuals that were physically active were classified as “healthy” agers. The remaining 98 were classified as “rapid” agers as they displayed poor walking ability despite being chronologically younger than the healthy agers (Breitbach et al., 2019; Figure 1a). Body bone mineral density and percentage lean body mass did not change significantly between these two groups suggesting the walking differences are not solely due to motor function (Figure S1). Multiple measures such as gait, function, mental status, strength, activity, and comorbidity index were also included in our study. Phenotypic age is an effective predictor of overall health risks and it is strongly associated with the chronological age (Fulop et al., 2010). However, one of the unique features of the ‘SOLVE‐IT’ study is that the biologically aged individuals are readily distinguishable from chronologically aged individuals. In this study cohort, frailty, comorbidities, impaired cognitive ability (defined by poor Montreal Cognitive Assessment scores), and higher body mass index (BMI) are negatively associated with chronological age and more prevalent in rapid agers. Such an inverse association of biological age and chronological age, as demonstrated in this cohort, offers an advantage in delineating specific signatures of healthy aging (Figure 1b). It is to be noted that incidence of declining organ functions such as heart, kidney, and liver failures, as well as, cancer was alike in both rapid and healthy agers (Figure 1b), thereby strengthening the unique appropriateness of the cohort for identification of markers associated with functional aging.

We used serum samples for our study since it is minimally invasive, affordable, and has reduced overall risk for patients. In addition, several recent studies with heterochronic parabiosis, as well as blood/serum transfusions in animal models suggest that systemic circulating factors in blood can accelerate biological age or act as geroprotectors (Pluvinage & Wyss‐Coray, 2020). To define the molecular fingerprint associated with biological aging, we performed high‐resolution metabolomics by ultrahigh‐performance liquid chromatography–tandem mass spectroscopy (UPLC–MS/MS). A total of 1327 serum metabolites were identified that belonged to nine different super pathways as defined by KEGG analysis. Majority of the identified metabolites were lipids (32%), followed by xenobiotics (17%) and amino acids (16%). The identified metabolite super pathways were further summarized into sub‐pathways. Long‐ and medium‐chain acylcarnitines (40), fatty acid dicarboxylate (31), sphingomyelins (29), diacylglycerols (29), and lysophospholipids (25) were predominant groups among lipids. Leucine, isoleucine and valine metabolism (33), arginine and proline metabolism (23), tryptophan metabolism (23) and methionine, cysteine, S‐Adenosyl methionine (SAM), and taurine metabolism (22) were some of the sub‐pathway profiles for amino acid metabolism that were identified (Figure 1c). The interrelationship between the identified metabolites revealed 1481 highly correlated (Spearman's *ρ* > 0.8) “metabolite pairs.” Over 200,000 significant correlations were observed among metabolite pairs, with 33,763 lipid–lipid pairs and 23,761 lipid–amino acid pairs. Interestingly, choline derivatives of long and very long‐chain free fatty acids (16–22 carbons) displayed remarkably high correlations. The identified fatty acylcholines (36) had a minimum *ρ* > 0.606; for example, palmitoylcholine (16:0) and steroylcholine (18:0) showed an exceptionally high correlation *ρ* = 0.94 (Figure 1d), suggesting a controlled acylcholine synthesis irrespective of heterogenous traits. Overall, our metabolomic analysis from this cohort generated a rich set of metabolite data that included several metabolic pathways.

### 2.2 Differential metabolome pattern associated with biological age

**FIGURE 2:**
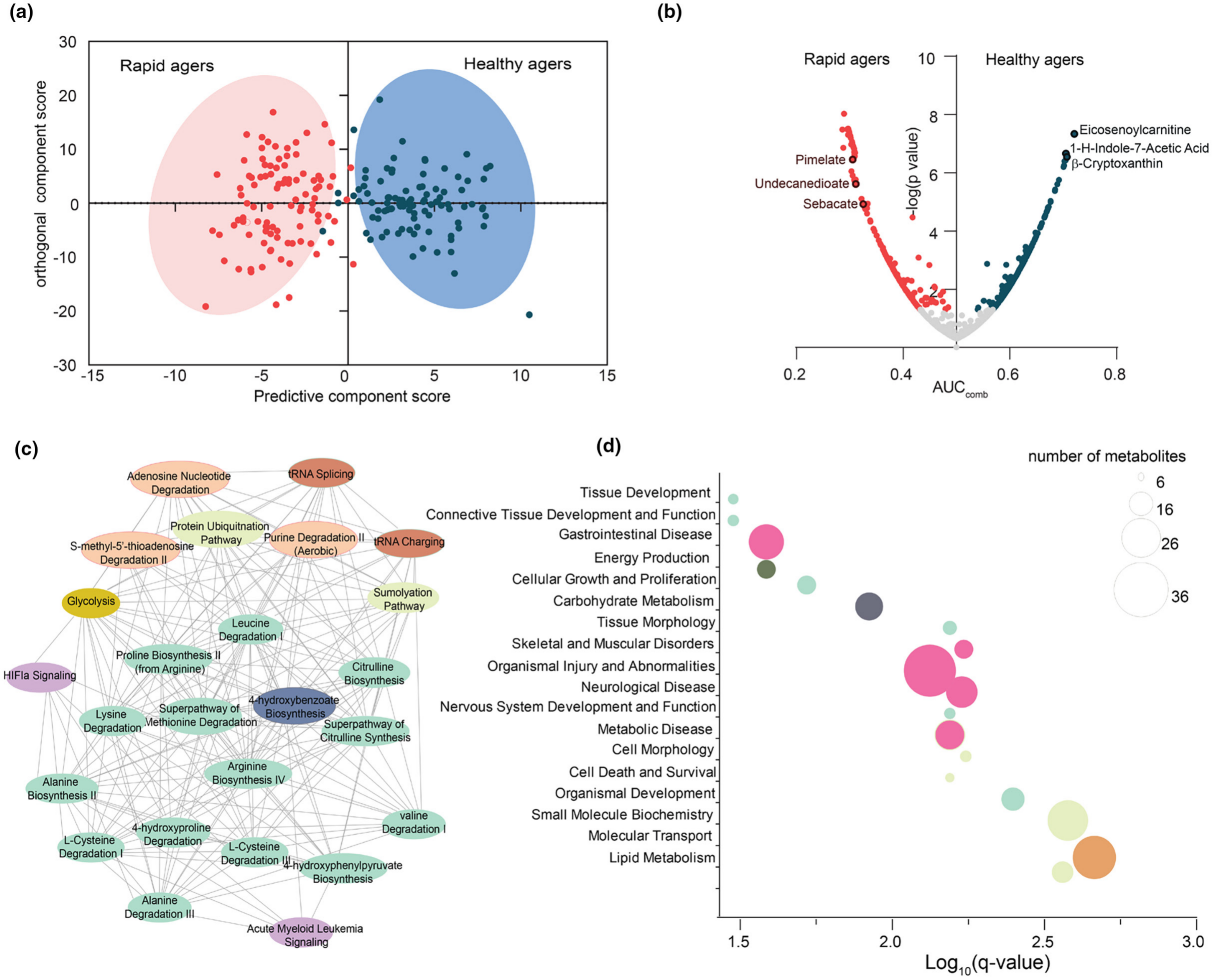
Metabolomic differences between healthy and rapid agers. (a) Difference in metabolomic profiles between healthy and rapid agers as evidenced from Orthogonal partial least square‐discriminant analysis (OPLS‐DA) score plot. (b) Receiver operating curve analysis (ROC) was performed to identify metabolites associated with healthy and rapid agers. Metabolites with AUC_comb_ (combined area under the curve) value >0.5 and adjusted *q* < 0.05 were predictive of healthy agers, whereas metabolites with an AUC_comb_ < 0.5 and *q* < 0.05 were predictive of rapid agers*; grey spots* represent non‐significant metabolites (c) Pathway‐enrichment analysis was performed using Qiagen Ingenuity Pathway Analysis (IPA). Network of enriched pathways in rapid agers shows alterations in amino acid (AA) biosynthesis. (d) Major disease and biofunction pathways associated with predictors of rapid agers are depicted in the bubble plot. Pathways are represented in *y*‐axis and the size of the bubble indicates the number of metabolites identified in each pathway. The *q‐*values were obtained following Benjamini–Hochberg correction of *p‐*values.

In order to test whether there are any differences between the metabolome of the healthy versus rapid agers, we used orthogonal partial least square‐discriminant analysis (OPLS‐DA), a multivariate supervised classification method with sevenfold cross validation consisting of 200 iterations in each round. Being a supervised dimension reduction analysis, OPLS‐DA, enables the reduction of complexity of the data and recognizes the patterns, trends, and interactions between metabolites. OPLS‐DA analysis produced a model with *R* ^2^ (cumulative) = 0.76, *Q* ^2^ (cumulative) = 0.40 with a predictive power of 95.9%, fisher *p*‐value = 1.45 × 10^−45^ and a root mean square cross validation error RMSE = 0.386. The model separated healthy and rapid agers demonstrating a clear difference in the metabolome associated with biological age (Figure 2a).

Receiver operating characteristic (ROC) curve analysis is a robust classification tool used to determine if an individual metabolite can distinguish between groups while accounting for confounding factors. To identify metabolites that are associated with healthy and rapid agers, we used ROC analysis based on a logistic regression model. Some metabolites were predictive of healthy agers [area under the curve (AUC) value for healthy aging ROC curve, ROC_AUC_HA_ > 0.5], and some were predictive of rapid agers [AUC value for rapid aging ROC curve, ROC_AUC_RA_ > 0.5]. We combined these ROC_AUC scores to create a single‐variable AUC_comb_ (see methods). Metabolites with AUC values more than 0.5 were considered indicators of healthy agers, while less than 0.5 were associated with rapid agers. There were 331 metabolites that significantly distinguished healthy and rapid agers, with 125 metabolites as predictors of healthy aging and 206 as predictors of rapid aging (*q*‐value <0.05) (Figure 2b). Eicosenoylcarnitine (C20:1), an acylcarnitine was one of the most influential metabolites in discriminating healthy agers from rapid agers (AUC_comb_ = 0.72). Beside acylcarnitines, healthy agers were also distinguished by β‐cryptoxanthin (AUC_comb_ = 0.70), a precursor of vitamin A, important for general growth, development, and immune response. Gut microbiome‐metabolite, 1H‐indole‐7‐acetic acid (AUC_comb_ = 0.70) was also elevated in healthy agers. In contrast, dicarboxylic fatty acids (DCAs) such as, pimelate (C7) (AUC_comb_ = 0.30), suberate (C8), sebacate (C10) (AUC_comb_ = 0.33), and undecanedioate (C11) (AUC_comb_ = 0.31) were elevated in rapid agers. Similarly, glutamate and mannose also elevated in rapid agers (AUC_comb_ = 0.33) (Figure 2b, source data).

To understand biological pathways associated with either healthy or rapid agers, we analyzed metabolites that significantly separated the two groups at *p*‐value <0.05, using Ingenuity Pathway analysis (IPA). We found that hypoxia‐inducible factor 1‐alpha (HIF1α) signaling, 4‐hydroxyproline degradation, adenosine nucleotides degradation, and citrulline metabolism were mainly associated with rapid biological age (Figure 2c). Consistent with our results, other studies have shown that upregulated HIF1α signaling impairs mitochondrial biogenesis and accelerates aging. Sirtuins are the main class of enzymes that destabilize HIF1α thereby promoting mitochondrial health during aging (Yuan et al., 2016). Similar to these reports, healthy agers positively associated with sirtuin signaling pathways, generally linked to longevity (Figure S2). Choline degradation, carnitine metabolism, and gamma‐glutamyl cycle were some of the other pathways associated with healthy agers (Figure S2). In addition to general metabolic pathways, IPA was used to provide disease and biofunction predictions. Disease/injury associated metabolites were related to gastrointestinal disease, skeletal and muscular disorders, organismal injury, and neurological diseases (Figure 2d). These data suggest that certain comorbidities may exhibit metabolic profiles which may indicate underlying conditions, although they were not clinically observed in rapid agers at the time of sample collection. Collectively, these results show that metabolites and associated pathways can differentiate healthy and rapid biological agers.

### 2.3 Balance of fatty acid oxidation pathways predicts healthy agers

**FIGURE 3:**
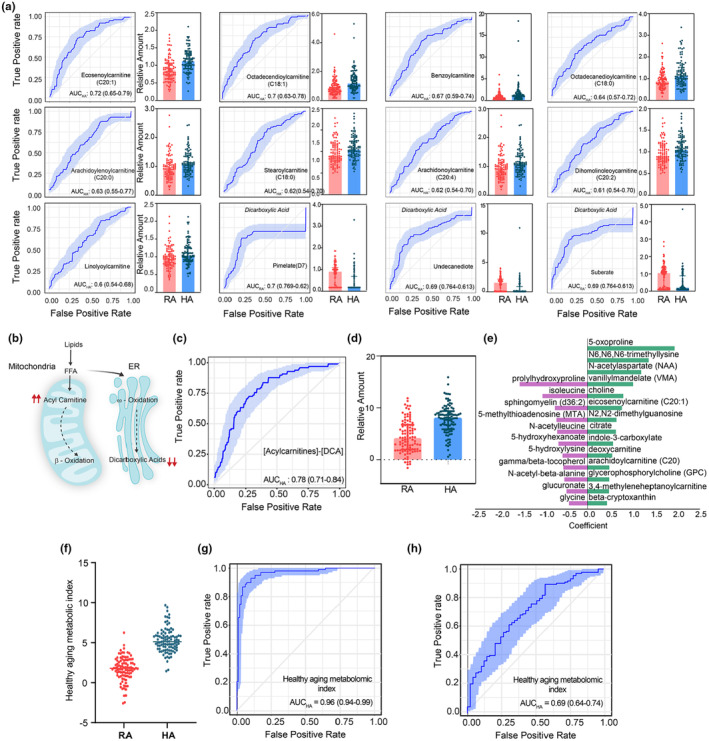
Identification of Healthy Aging Metabolic (HAM) Index. (a) ROC curves for acylcarnitines and dicarboxylic acids (DCA) and their distribution profile in healthy (HA) and rapid agers (RA). Acylcarnitines are increased in healthy agers whereas dicarboxylic acids are decreased. AUC_HA_ = AUC value of ROC curve generated for HA (RA as controls); AUC_RA_ = AUC value of ROC curve generated for RA (HA as controls). (b) Free fatty acids (FFA) released from complex lipids are substrates for fatty acid oxidation pathways; β‐oxidation (primarily in mitochondria) and ω‐oxidation (microsomes, ER). The FFA are shuttled into mitochondria via acylcarnitines; consequently, increased levels of acylcarnitines indicate active β‐oxidation. Similarly, DCAs are intermediary products of ω‐oxidation and can serve as indicators of active ω‐oxidation. (c) ROC curve and (d) distribution of the difference in acylcarnitines and DCAs in healthy and rapid agers. (e) List of metabolites and its coefficients identified from the model. Healthy Aging Metabolic (HAM) index, was developed using the model parameters. (f) HAM index showed a significant difference between rapid agers and healthy agers (g) high predictive power (AUC_HA_ = 0.95). (h) ROC analysis in validation cohort, WRAP, showed an AUC value of 0.68 in predicting healthy agers from rapid agers (above 70 years, could walk more than 10 mins without stopping at least once in a week versus rarely walks more than 10 min without stopping).

Next, we sought to identify specific metabolite signatures that can serve as potential indicators for healthy aging. Acylcarnitines, especially long chain acylcarnitines and dicarboxylic acids (DCAs) were the two important classes that were identified both in the OPLS‐DA and ROC analyses (Figure 3a). Acylcarnitines play a major role in regulating lipid metabolism by shuttling fatty acids into mitochondria. Under physiological conditions, oxidation of long‐ and medium‐chain fatty acids is primarily carried out by mitochondrial β‐oxidation (Figure 3b). We observed nine acylcarnitines, mostly long‐chain forms with ROC_AUC_HA_ values 0.6–0.72 (Figure 3a). An alternate, subsidiary pathway to β‐oxidation is ω‐oxidation occurring in the endoplasmic reticulum (ER) microsomes (Miura, 2013). ω‐oxidation of fatty acids generates dicarboxylic fatty acids (DCAs) and is an alternate pathway used when mitochondrial β‐oxidation is impaired (Wanders et al., 2011) (Figure 3b). Our analysis detected three important DCAs: pimelate, undecanediote, and suberate with ROC_AUC_RA_ values 0.6–0.7. In healthy agers, the acylcarnitines levels were higher than in rapid agers suggesting that β‐oxidation is predominantly active in the former. On the other hand, rapid agers showed higher levels of DCAs compared to healthy agers, indicating increased ω‐oxidation (Figure 3a). The predictive power obtained from the difference in acylcarnitines and DCAs improved by 0.06 compared to the best individual predictor (eicosenoylcarnitine) (Figure 3c,d). These results suggest a balance between β‐oxidation and ω‐oxidation pathways can potentially influence biological age.

### 2.4 Identification of Healthy Aging Metabolic (HAM) index

Aging is a complex process that cannot be comprehended through one metabolic pathway or a metabolite class. Indeed, several reports have implicated the roles of multiple pathways affecting the aging process. For example, dysregulation of the carnitine shuttle and vitamin E pathways have been associated with frailty (Rattray et al., 2019) whereas, tryptophan metabolism, particularly, kynurenine pathway is implicated in age‐related chronic inflammation and memory impairment (Sorgdrager et al., 2019), as well as muscle aging (Janssens et al., 2022). Therefore, we hypothesized that a combination of metabolites from different pathways could possibly be a better molecular fingerprint for biological age, rather than a single metabolite/pathway. The combinatorial approach can overcome the moderate predictive power presented by the individual metabolites. However, modeling from a small cohort such as SOLVE‐IT can lead to significant overfitting. Therefore, we decided to model rapid and healthy aging using SOLVE‐IT and validate the model with an external cohort. We used a previously reported larger cohort, WRAP (Wisconsin Registry of Alzheimer's Patients), as the validation cohort. The WRAP cohort consists of participants enrolled in midlife (mean age 54) and longitudinally followed (Johnson et al., 2018), includes a variety of health indices and has associated metabolomics data with a similar number of identified metabolites, making it an ideal cohort to use as test dataset.

The metabolite data from the WRAP cohort consist of 1296 metabolites. In order to identify a panel of metabolites that are better predictors of healthy biological aging we chose all known metabolites that are common to both datasets, SOLVE‐IT and WRAP. Next using the SOLVE‐IT dataset, we performed OPLS‐DA analysis and selected all metabolites with variable importance of the projection (VIP) score >1 to fit a linear regression model. We used LASSO regression method with 10‐fold cross validation with 1000 bootstrapping steps in each validation. LASSO regression model eliminates the collinear variables and retains only the significant variables (*p* < 0.05). The final model retained a panel of 25 metabolites with a Pearson's *r* = 0.74 and *p* < 0.0001 (Figure 3e). The model consisted of metabolites primarily related to fatty acid metabolism, the TCA cycle, and amino acids and it strongly predicted healthy biological agers. Importantly, based on the model values we derived a healthy aging indicator, “Healthy Aging Metabolic (HAM) Index.” The HAM index was significantly different between the healthy agers and rapid agers (*p* < 0.0001) and showed a ROC_AUC_HA_ value of 0.95 in identifying healthy agers (Figure 3f,g). HAM index showed a moderate correlation with other biological indices such as frail score and comorbidity sum (Table S1). HAM index also showed significant predictive power in differentiating SOLVE‐IT cohort population—classified based on the frailty and comorbidity. When grouped based on frailty, HAM index's AUC value was 0.72 in predicting frail group from the non‐frail group (Figure S3a). On the other hand, HAM index showed an AUC‐ROC value of 0.75 in differentiating seniors with less than four comorbidities from those with ≥4 comorbidities (Figure S3b). The HAM index outperformed other indices such as frailty index, gait speed, and MOCA score in predicting healthy agers from rapid agers (Figure S3c).

We then sought to validate the effectiveness of the HAM index using metabolomics data from the WRAP cohort. In this group, the HAM index demonstrated an AUC value of 0.68 in predicting healthy individuals over the age of 70 who can walk outside their home for more than 10 min without stopping at least once per week, compared to those who rarely or never walk outside (Figure 3h). Additionally, the HAM index showed a significant AUC value in predicting individuals over the age of 45–65 who can walk outside their home for more than 10 min without stopping at least once per week versus those who rarely or never walk outside (Figure S3d–h). The HAM index also showed a moderate but highly significant negative correlation with comorbidity in the WRAP cohort (Figure S3i) with a correlation coefficient of −0.15 and a *p*‐value of 2.8 × 10^−14^. As the rapid agers are chronologically younger than the healthy agers, it is possible that the HAM index could simply reflect the effect of aging, rather than the rate of aging, in the sample groups. To test this, we correlated chronological age with the HAM index in the validation cohort, WRAP. As shown in the figure (Figure S4a), the HAM index correlated poorly with chronological age, with an *r*‐squared value of 0.01. We also tested the ability of the HAM index to differentiate between people over 70 who rarely walk outside (equivalent to rapid agers), and people aged 60–70 who walk at least once a week (equivalent to healthy agers). The HAM index showed a an AUC of 0.602 in ROC analysis (Figure S4b). The frequency of non‐walkers and walkers by age from the WRAP cohort is described (Figure S4c). These results strongly suggest that the effects observed here are not merely the effect of chronological aging, but rather indicate healthy aging. This predictive power from a combination of metabolites indicates that several different pathways are involved in maintaining a healthy biological age.

### 2.5 SASP markers associated with rapid agers

**FIGURE 4:**
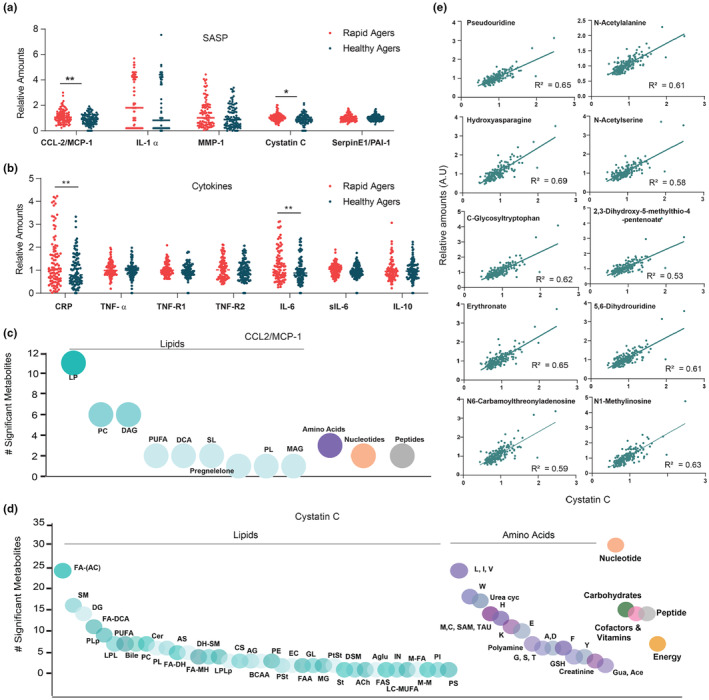
Senescence‐associated secretome linked with biological age. (a, b) Distribution of SASP and proinflammatory factors in healthy and rapid agers. CCL2‐/MCP‐1, cystatin C, CRP, and IL‐6 levels were increased in rapid agers compared to healthy agers. Bonferroni corrected *p < 0.05; **p < 0.01. (c, d) The number of metabolites significantly associated with each super pathway are represented as bubble plots for (c). CCL2/MCP‐1 and (d) cystatin C. Lipids were further divided into its sub pathways in both (c and d). Amino acids were further classified in (d) metabolites with FDR < 0.2 for partial Spearman's *ρ* controlling for age and gender were designated as significantly correlated metabolites. (e) Scatter plot displaying the top 10 highly correlating metabolites with cystatin C (*R* ^2^ > 0.5).

Circulating factors such as senescence‐associated secretory phenotype (SASP) and pro‐inflammatory markers can reflect the state of aging cells. Senescence‐associated “secretome” could be a valuable marker for aging and age‐associated diseases. With this in mind, we wanted to investigate age‐associated proinflammatory markers in the context of biological age. To test this, we measured multi‐analyte SASP and proinflammatory markers in serum using Luminex High Performance Assay. The list of SASP and proinflammatory markers examined were based on evidence from several studies (Table S2). Interestingly, SASP markers CCL‐2/MCP‐1 and IL‐6 were found to be elevated in rapid agers compared to healthy agers. In addition, cystatin C (Evans et al., 2023) and C‐reactive protein (CRP) were also elevated in rapid agers (Figure 4a,b). These data suggest that a subset of SASP and proinflammatory factors track with increased biological age independent of chronological age.

Next, we examined the metabolomic profiles associated with these elevated SASP and proinflammatory markers. As shown in Figure 4c and source data, CCL‐2/MCP‐1 was associated with 57 metabolites (FDR‐correlated *p* < 0.2) with majority (56%) contributed by lipids, particularly, lysophospholipids (11), diacylglycerols, (6) and phosphatidyl glycerol (6). In contrast to CCL‐2/MCP‐1, cystatin C showed positive association with metabolites that belonged to both lipids (175) and amino acids (150) (Figure 4d, source data). A panel of serum metabolites that displayed remarkable correlation with cystatin C is shown in Figure 4e. Interestingly, the levels of CCL‐2/MCP‐1 and cystatin C levels were random among the age groups and did not influence one another as suggested from Spearman's *ρ* with a *p*‐value >0.05. Tryptophan (4), tyrosine (4) metabolism, fibrinogen cleavage peptide (7), vitamin E metabolism, and androgenic steroids (7) showed positive association with CRP whereas ceramides (5) were negatively associated with CRP. Cleaved fibrinogen products, as well as, higher levels of tryptophan metabolism products such as kynurenine and indole‐3‐carboxylates suggest chronic inflammation in rapid agers (Table S2). Metabolites associated with IL‐6, particularly DSGEGDFXAEGGGVR, fibrinopeptide B (1–13), ADSGEGDFXAEGGGVR, and fibrinopeptide A (3–16) also support the prevalence of a low‐grade inflammation with rapid biological age (Lustgarten & Fielding, 2017).

In order to understand the relationship between metabolites/metabolic pathways and circulating secretory factors in aging, we looked for common metabolites that were associated with secretory factors and biological age groups. Five classes of metabolites‐ acylcarnitine, oleoyl/linoleoyl glycerol phosphocholine, carotene diol, γ‐glutamyl glutamine, and nicotinamide that were strongly associated with healthy aging were found to be negatively associated with Cystatin C, MCP‐1, CRP, as well as, IL‐6 (Figure S5). Similarly, DCAs, key products of ω‐fatty oxidation that were strongly associated with rapid agers were positively associated with SASP markers, MCP‐1 and IL‐6 (Figure S6). Taken together, our data reports a SASP/inflammatory‐metabolome network for biological age.

### 2.6 Metabolites display sexual dimorphism independent of biological age

**FIGURE 5:**
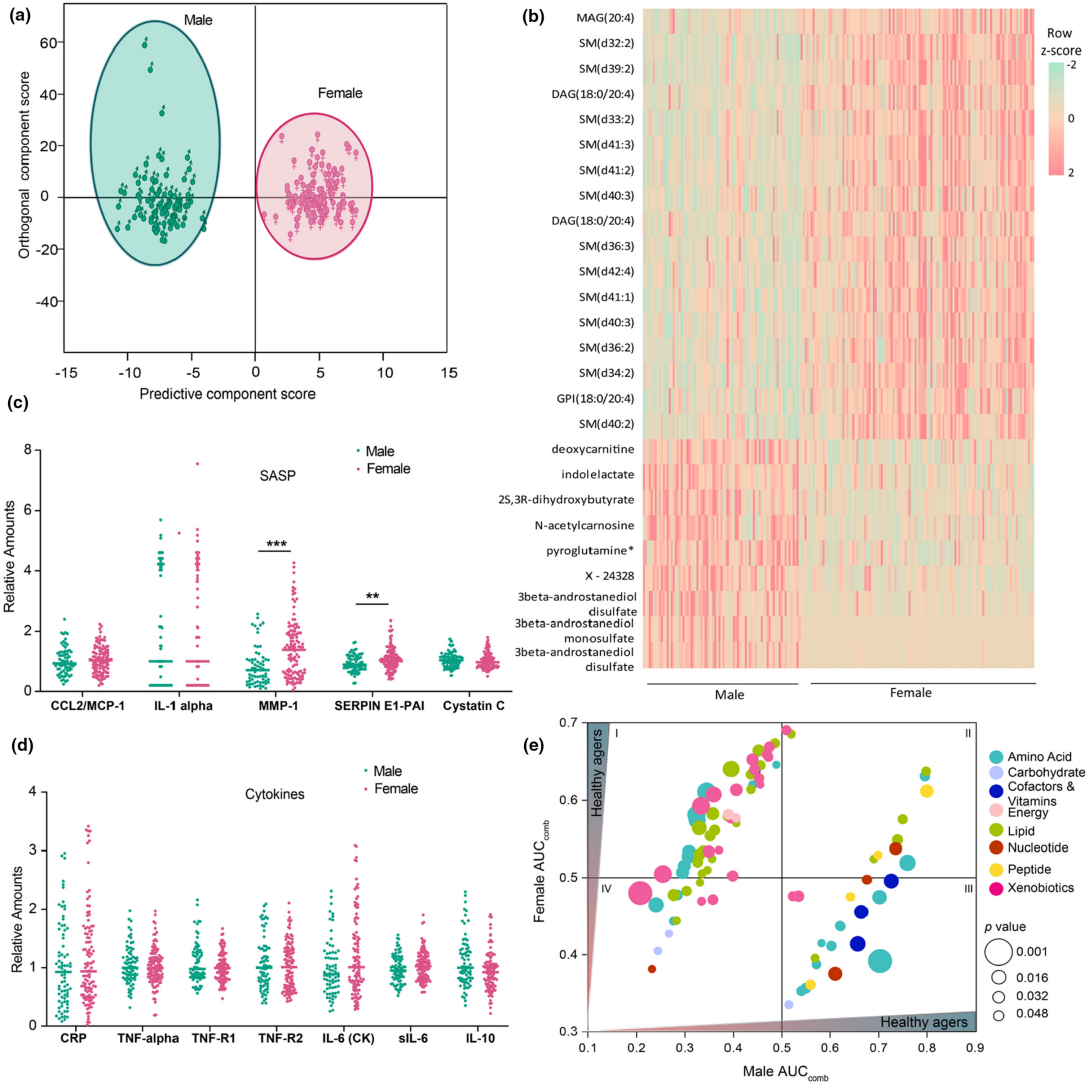
Sexual dimorphism in aging. (a) OPLS‐DA score plot showing the class separation between males and females in the study population. The separation along predictive axis indicates metabolic distinction between the two groups. (b) The top two percentile metabolites identified from the OPLS‐DA analysis are represented as a heat map. A dot plot distribution of (c). SASP and (d) proinflammatory factors in male and female population. Bonferroni corrected **p < 0.01; ***p < 0.001 (e) bubble plot showing the AUC_comb_ of metabolites that significantly differ between male and female groups in predicting healthy agers. AUC_comb_ > 0.5 values = healthy agers, AUC_comb_ < 0.5 values = rapid agers. Metabolites are color coded based on their super pathways and the sizes represent the *p*‐value. Cluster I: metabolites elevated in female‐healthy agers but decreased in male‐healthy agers. Cluster II: metabolites elevated in healthy agers of both genders. Cluster III: metabolites that were elevated in female‐ and male‐rapid agers. Cluster IV: metabolites that were decreased in female‐rapid agers but increased in male‐rapid agers. The ROC curves of metabolites were compared using bootstrap method. Significant metabolites (*p* < 0.05) were plotted.

Several factors can influence biological age, one such is gender. It is well known that life expectancies for women are usually higher than men (Austad & Fischer, 2016; Bronikowski et al., 2011). Here we probed the metabolome of our study cohort to understand the implications of sexual dimorphism in biological aging. Consistent with the previous reports (Darst et al., 2019), there was a clear metabolomic difference between the males and females in the SOLVE‐IT cohort (Figure 5a, Figure S7). Among females, sphingomyelins were significantly increased compared to males. Similarly, the levels of one of the major endocannabinoids, arachidanoyl glycerol and its precursor stearoyl arachidanoyl glycerol were higher in females (Figure 5b). On the other hand, as expected in males, the male hormone, androgen‐derived metabolites such as androstenediol disulfate (1), androstenediol monosulfate, (2) were found to be elevated. Examining the SASP and proinflammatory factors, it was clear that matrix metalloproteinase (MMP‐1) and plasminogen activator inhibitor‐1 (PAI‐1) were significantly increased in females compared to males. Taking our previous results into consideration (Figure 4a), we observed that irrespective of the gender, both CCL‐2/MCP‐1, IL‐6, CRP, and cystatin C were unaltered suggesting that biological aging‐associated secretome may not be influenced by gender (Figure 5d).

Next, we sought to understand the impact of gender in biological age‐associated metabolic signatures (Figure 5e). For this, we compared the ROC curves of males versus females in predicting healthy and rapid agers. This enables a direct comparison between the sexes by factoring age groups but without compromising the statistical power of the analysis. The analysis identified four groups of metabolites: Cluster I‐ metabolites that were elevated in *male rapid* agers, but lower in *female rapid* agers (male AUC_comb_ < 0.5 and female AUC_comb_ > 0.5, upper left quadrant); Cluster II‐ metabolites elevated in *healthy* agers of both the sexes (male AUC_comb_ > 0.5 and female AUC_comb_ > 0.5, upper right quadrant); Cluster III‐ metabolites elevated in *male healthy* and *female rapid* agers (male AUC_comb_ > 0.5 and female AUC_comb_ < 0.5, lower right quadrant), and Cluster IV‐ metabolites elevated in *rapid* agers of both the sexes (male AUC_comb_ < 0.5 and female AUC_comb_ < 0.5, lower left quadrant). A total of 113 metabolites were identified, with 57 metabolites mapping to Cluster 1 (*male rapid* agers, linked to *female healthy* agers). Six acetylated metabolites namely, N2‐acetyl, N6‐methyllysine, N2‐acetyl, N6, N6‐dimethyllysine, N‐acetylcitrulline, N‐acetyl phenylalanine, N‐acetylarginine, and N‐acetyl‐3‐methylhistidine belonged to Cluster I. Likewise, very‐long‐chain (C > 22) acylcarnitines such as nervonoylcarnitine, cerotoylcarnitine, behenoylcarnitine, and ximenoylcarnitine correlated remarkably well with female healthy agers but not with male healthy agers. However, long‐chain acylcarnitines (18 ≤ C ≤ 22) were associated with both, male and female healthy agers. This suggests that even though acylcarnitines are universal markers of aging (Rattray et al., 2019), gender can have a significant impact. Similarly, some *male healthy* aging‐associated metabolites (24) were identified with *female rapid* agers. For example, oxidized methionine, methionine sulfone showed the highest difference in the AUC_comb_ values with 0.70 in males and 0.39 in females. A few amino acid metabolites such as ornithine, 5‐(galactosylhydroxy)‐L‐lysine, C‐glycosyltryptophan, 3‐methyl glutaryl carnitine, cystathionine, dimethylguanidino valeric acid, hydantoin‐5‐propionate, and hydroxyasparagine were also identified with healthy agers in males but not with females.

We identified 16 metabolites that were associated with healthy agers in both, males and females but with significant differences in their power of association (Cluster II). The top metabolites in this group were long‐chain acylcarnitines like octadecanedioylcarnitine, octadecenedioylcarnitine, which are strong predictors of *male healthy* agers but weak predictors of *female healthy* agers. Similarly, gamma‐glutamylcitrulline and S‐methyl methionine could predict the *male healthy* agers with an AUC_comb_ value of 0.80 but this value was reduced to 0.61 and 0.63, respectively, in *female healthy* agers. On the other hand, levels of two major glycolytic metabolites; glucose and pyruvate were strong predictors of *male rapid* agers but their predictive power was significantly lower for *female rapid* agers (Cluster IV) (Figure 5e, source data). Interaction plot shows the AUC_comb_ of metabolites that significantly differ between male and female groups in predicting healthy agers (Figure S8a). Overall, these results suggest a decisive role of sexual dimorphism in the metabolism associated with aging. Of note, none of the biomarkers from the HAM index was affected by gender differences, pointing to the robustness of the HAM index in predicting healthy agers.

### 2.7 Metabolites associated with smokers

**FIGURE 6:**
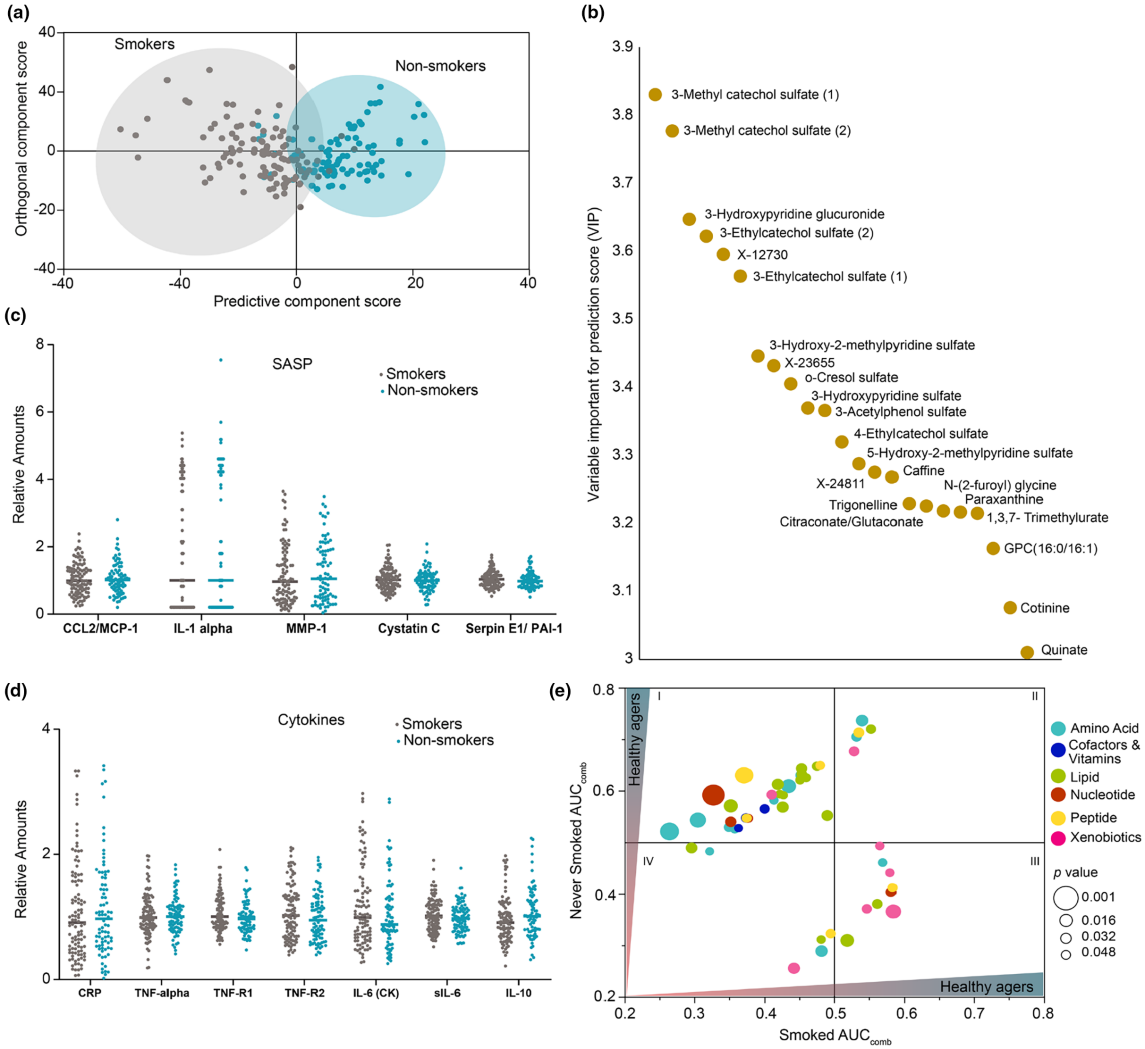
Effect of smoking on biological aging. (a) OPLS‐DA score plot shows a moderate separation of smokers and nonsmokers. (b) The top predictive metabolites that differentiate smokers and nonsmokers are represented as a dot plot. Most metabolites belonged to xenobiotics. Influence of (c, d) SASP and proinflammatory markers in smoking and nonsmoking groups are shown as dot plots. (e) Differences in AUC of metabolites among the smoking and nonsmoking population and its influence on predicting healthy aging is shown as a bubble plot.

Biological aging can be influenced by choices such as smoking. Cigarette smoke produces numerous (~4000) compounds with varying levels of toxicity and is known to increase the risk of COPD, cardiovascular disease and other age‐related diseases. Previous studies have established differences in metabolome of smoker and never‐smokers individuals (Hsu et al., 2017). However, the effect of smoking on the metabolites associated with biological age remains unexplored. Therefore, we analyzed the effect of smoking on the metabolome of the SOLVE‐IT cohort. Our OPLS‐DA analysis demonstrated a moderate separation between the individuals that smoked and those that never smoked (Figure 6a). This separation was predominantly due to xenobiotics related to benzoates and caffeine metabolism. Some of the major metabolites in this list include, 3‐methyl catechol sulfate(s), 3‐ethyl catechol sulfate(s), and caffeine (Figure 6b). It is important to note that about 91% of the “smokers” cohort had quit smoking (average years since they quit was 31 years). We did not observe any changes in both SASP and proinflammatory markers (Figure 6c,d) suggesting a “prior smoking status” did not induce prolonged low‐grade inflammation.

We then compared the impact of smoking on metabolites associated with biological age. Overall, 67 metabolites were significantly altered by the smoking status in the context of biological aging. Cyclic AMP (cAMP) was strongly associated with healthy agers among the smokers but not the never‐smokers. cAMP is known to slow aging process by binding to sirtuins 1 and 3 (Wang et al., 2015). Interestingly, it has been reported that cAMP levels increase during smoking (Wong et al., 2018). Therefore, one possible explanation of our data is that the healthy biological aging of some of the smokers in our study cohort may be linked to the increase in their cAMP levels. Similarly, choline, urea, and guanidinosuccinate were strongly associated with the healthy agers of smokers population but they were fairly distributed among healthy and rapid agers of non‐smokers. Metabolites such as trigonelline, 3‐ethylcatechol sulfate, and methyl 3‐catechol sulfate were elevated among healthy agers of “non‐smokers” population but were not observed in the “smokers” population (Figure 6e, source data). These metabolites are usually from food; but, it is postulated that smoking can enhance its biological conversion (Gu et al., 2015; Wang et al., 2018). Differences in AUC of metabolites among the smoking and nonsmoking population and its influence on predicting healthy aging is shown as an interaction plot (Figure S8b). Our results indicate that trigonelline, 3‐ethylcatechol sulfate, and methyl 3‐catechol sulfate may not be affected long‐term by smoking. Taken together, our results suggest that prior smoking does have a long‐term shift in the metabolites, but its effect on biological age is only moderate.

### 2.8 Computational discovery of causal relationships

**FIGURE 7:**
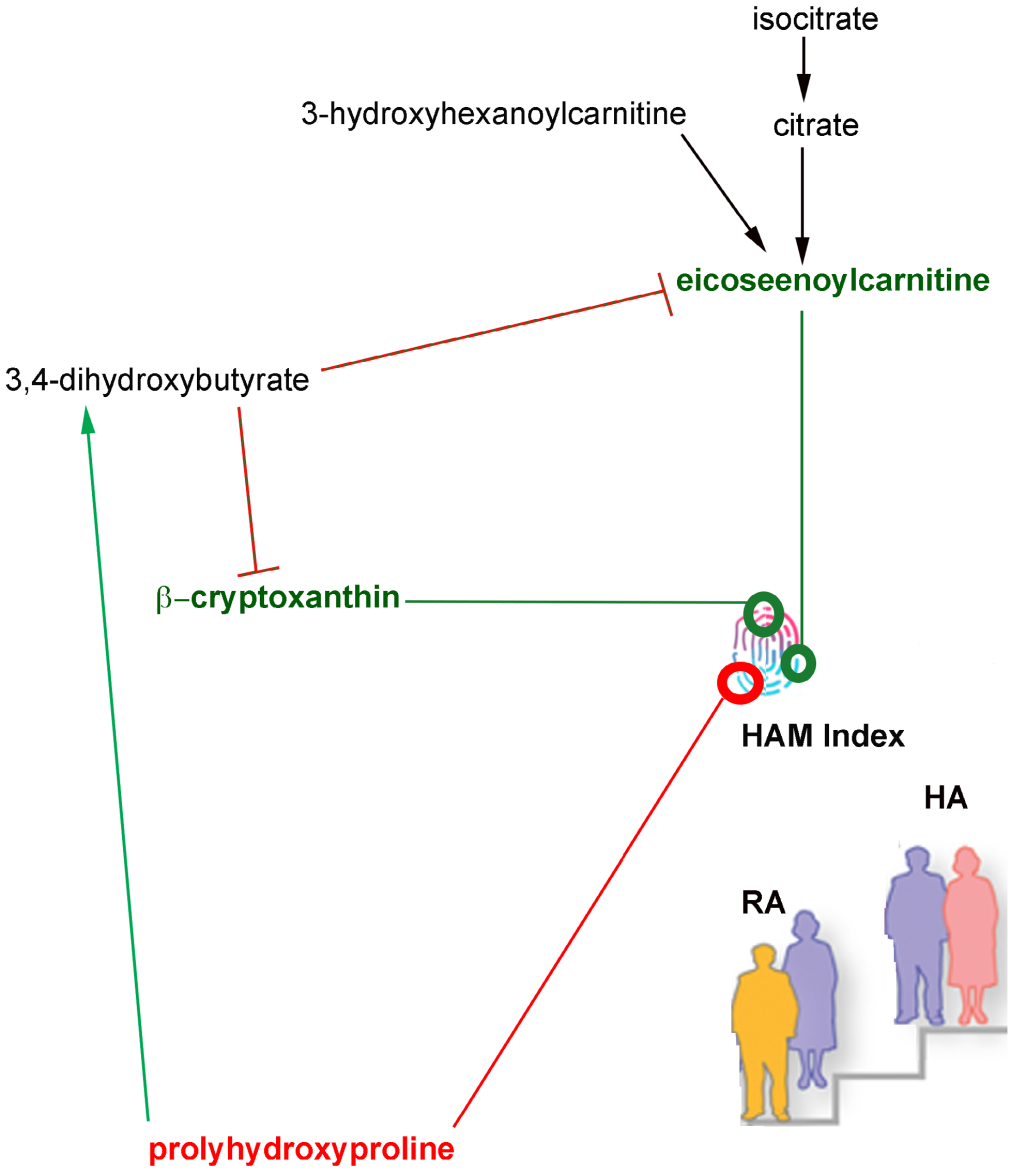
Causal metabolites for biological aging. Eicosenoylcarnitine and β‐cryptoxanthin were positively causal to the HAM index, whereas prolyhydroxyproline had a negative impact on the HAM index. Other metabolites, such as 3,4 dihydroxybutyrate was also seen to negatively impact β‐cryptoxanthin and eicosenoylcarnitine. Interestingly, prolyhydroxyproline was identified to positively influence 3,4 dihydroxybutyrate, suggesting cross talk between these metabolites underlying biological aging.

Discovering predictive biomarkers coupled with identifying putative causal factors can propel the field forward by allowing translational experiments and/or clinical interventions that can test a functionally prioritized set of hypotheses. Therefore, we explored causal discovery with the PC‐Stable algorithm using a threshold value of *α* = 0.05 (used in conditional independent tests within the algorithm) and a bootstrap value of 50. Our data suggests a limited set of relationships (Figure 7). The causal network shows many inferred relationships between measured clinical variables (Figure S9). There are three metabolites that appear to causally impact the HAM index—two strongly positively (β‐cryptoxanthin and eicosenoylcarnitine) and one weakly negatively (prolylhydroxyproline). It is noteworthy that the other metabolites used to compute HAM index are not directly causal influencers of the HAM index. This may suggest that their contributions are being accounted for by other variables in the system. Taken together, our data highlight key metabolites that need to be further examined for their impact on biological aging.

## 3 DISCUSSION

Biological age captures one's physical and functional ability and is a more accurate determinant of healthspan than chronological age. A few aging indicators using “omics” data have been identified in recent years, but the most accurate way to assess biological age remains unclear (reviewed; Panyard et al., 2022). Emerging studies analyzing metabolomics associated with age‐related diseases, mortality, and longevity have provided novel insights (Hornburg et al., 2023; Wang et al., 2023). However, several of these studies have limited number of metabolites profiled or use chronological age to identify metabolites associated with aging.

In this study, we selected a cohort that offers a possible approach to distinguish chronological aging from biological aging. Our groups were separated by chronological age, with all rapid agers between 65–75 years of age and healthy agers over 75 years of age. To the best of our knowledge, this is the first study to use a cohort that has a negative relationship between biological and chronological aging. Although this is unusual in a normal population, this separation allows us to extract out the metabolic changes that *likely* occur during biological aging. We identified a panel of 25 metabolites (HAM index) that could predict healthy agers. The HAM index strongly predicted healthy biological agers and was not affected by the demographic and lifestyle factors such as gender, race, and smoking status (Figure S10). We also examined the SASP and cytokine/chemokine panel to identify markers of biological age. This approach provides a unique molecular fingerprint associated with rapid and healthy aging.

Acylcarnitines play a major role in regulating lipid metabolism by shuttling fatty acids into mitochondria. Under physiological conditions, oxidation of long‐ and medium‐chain fatty acids is primarily carried out by mitochondrial β‐oxidation. An alternate, subsidiary pathway to β‐oxidation is ω‐oxidation occurring in the endoplasmic reticulum (ER) microsomes. ω‐oxidation of fatty acids generates dicarboxylic fatty acids (DCAs) and is an alternate pathway used when mitochondrial β‐oxidation is impaired. In healthy agers, the acylcarnitines levels were higher than in rapid agers, suggesting that β‐oxidation is predominantly active in the former. Several reports in model organisms indicate the importance of fatty acid metabolism in maintaining healthspan and longevity (Hamsanathan et al., 2022; Mutlu et al., 2021). For example, overexpression of a β‐oxidation enzyme, dodecenoyl CoA delta isomerase in *Drosophila* extended lifespan (Lee et al., 2012). Similarly, in *C. elegans* lipid‐binding protein signaling increases mitochondrial β‐oxidation, decreases lipid storage and promotes longevity, thus implying the role of β‐oxidation in healthy aging (Ramachandran et al., 2019). On the other hand, rapid agers showed higher levels of DCAs compared to healthy agers, indicating increased ω‐oxidation. Our data indicate that a balance between β‐oxidation and ω‐oxidation pathways can potentially influence biological age. Whether the impairment of β‐oxidation or a preference for ω‐oxidation drives rapid aging needs to be further explored. There is a complex relationship between these patways, including evidence that supports upregulation of ω‐oxidation directly responsible for suppression of β‐oxidation, and possible lipid accumulation in the liver (Zhang et al., 2021). Other studies suggest that ω‐oxidation is an alternative to β‐oxidation during low‐carnitine conditions and is inefficient in the handling of reactive oxygen species (ROS) (Wanders et al., 2011). The enzyme monooxygenase cytochrome P450 involved in ω‐oxidation is one of the major producers of H_2_O_2_ in microsomes and could therefore possibly drive cellular oxidative stress (Johnson et al., 2015) and contribute to rapid aging. Taken together, this evidence supports the balance between fat oxidation pathways can determine one's biological age.

Eicosenoylcarnitine, an acylcarnitine with 20‐carbon acyl groups, was noted as being one of the most influential metabolites in discriminating healthy agers from rapid agers (AUC_comb_ = 0.72). Indeed, there have been other reports with an increase in eicosenoylcarnitine in healthy aging (Jarrell et al., 2020). β‐cryptoxanthin, a carotenoid found in fruits and vegetables, is positively associated with the HAM index, and potentially causal to healthy aging. Consistent with our finding, β‐cryptoxanthin has been associated with reduced risk of multiple age‐related diseases, including cancer, osteoporosis, and other degenerative diseases. Interestingly, lower serum levels of β‐cryptoxanthin were also observed in the MARK‐AGE cohorts and associated with increased risk of cognitive frailty (Rietman et al., 2019). There are few studies of β‐cryptoxanthin supplementation in humans—and these may show some promise. For example, supplementation with β‐cryptoxanthin increased bone mineral density in postmenopausal women and suppressed cytokine profile (Sugiura et al., 2016). However, most of the supplementation studies have very low power and have not examined for age‐associated pathologies yet (Kim et al., 2021). On the other hand, degradation of collagen is one of the pathologies identified with chronological aging (Mays et al., 1991). Hydroxyproline is a marker of bone loss and collage degradation. It has also been established as a marker of bone resorption. However, whether inhibition of such collagen degradation can promote healthy aging is not yet well‐understood. Further exploration of the connection between these metabolites and biological aging is needed.

The role of fixed factors, such as gender, in aging is well known. Our ROC analysis illustrated the influence of sexual dimorphism on aging, specifically very long chain acylcarnitines in female healthy agers. Very long chain acylcarnitines are present in peroxisomes and mediate β‐oxidation of very long chain saturated fatty acids (VLSFA). Peroxisomes are crucial organelles that govern cell aging by maintaining homeostasis of ROS and metabolic homeostasis (Titorenko & Terlecky, 2011). Increase of VLSFA carnitines in female healthy agers suggests active peroxisomal activity which may also aid in longer life expectancies. Supporting this, studies have shown that increased circulating levels of VLSFA reduce the risk of coronary heart disease (Lemaitre et al., 2018; Malik et al., 2015). Accumulation of oxidized proteins increase with chronological aging and it has been shown that gender differences influence the process of protein oxidation during aging (Reeg & Grune, 2015). Our results indicate that degraded products of oxidized proteins such as methionine sulfone are higher in healthy male agers, suggesting effective degradation and removal of accumulated oxidized proteins in healthy agers.

SASP factors are secreted by senescent cells that accumulate with chronological age and drive multiple age‐related pathologies (Childs et al., 2015). Recent reports have identified SASP and cytokine/chemokine signatures with aging and lifestyle interventions that improve health (Englund et al., 2021; Fielding et al., 2022; Schafer et al., 2020). Here we analyzed a spectrum of SASP and cytokine/chemokine factors for potential indicators of rapid biological aging. CCL‐2/MCP‐1 and cystatin C were significantly increased in the rapid agers compared to healthy agers. CCL‐2/MCP‐1 is a crucial component of the SASP in some senescent cell types and treatment with a senolytic (intervention to eliminate senescent cells) has been shown to significantly reduce MCP1 (Yousefzadeh et al., 2018). Additionally, MCP1 was found to be significantly higher in frail older adults compared to non‐frail adults (Yousefzadeh et al., 2018). In addition, our study explored SASP/cytokine–metabolome network. CCL‐2/MCP‐1 was strongly associated with several metabolites including two phospholipid classes (PLC): lysophospholipids and diacylglycerols. Consistent with this, lysophosphatidic acid, has been shown to upregulate CCL‐2/MCP‐1 levels (Lin et al., 2006). Similarly, the role of CCL‐2/MCP‐1 inducing PLC to produce diacylglycerol has been observed in a previous study (Bose & Cho, 2013), suggesting that MCP‐1 associated metabolites need to be further examined for their role in biological age.

Cystatin C is a protease inhibitor primarily involved in the pathology of renal dysfunction. Plasma cystatin C was recently identified as an effective marker in the assessment of kidney function compared to the traditional creatinine (Roos et al., 2007). In addition, cystatin C is associated with, cardiovascular diseases (Go et al., 2004), neurological disorders (Sundelöf et al., 2008), and cancer (Strojan et al., 2004). Considering the strong association of cystatin C with renal dysfunction, increased levels of cystatin C with rapid aging may suggest underlying renal dysfunction that could possibly be a consequence of senescence. Another interesting observation is the extremely high correlation of cystatin C with the various metabolites. For example, given the heterogeneous sample set, the R (Franceschi & Campisi, 2014) value of 0.69 between hydroxyaspargine and cystatin C is very rare among unrelated metabolites. This strong association is indicative of a close biological interaction between two biomolecules. To the best of our knowledge, the direct relationship between cystatin C and the highly correlated metabolites is not reported in literature. From this panel, C‐glycosyltryptophan, pseudouridine, O‐sulfotyrosine, N‐acetylthreonine, N‐acetylserine, N6‐carbamoylthreonyladenosine, and N6‐acetyllysine were previously reported to be associated with impaired renal function (Niewczas et al., 2017). Furthermore, IL‐6 and CRP, were also increased in rapid agers. The PolSenior study showed the correlation between the two cytokines and chronological age (Puzianowska‐Kuźnicka et al., 2016). Our study further indicates that IL‐6 and CRP may in fact be predictors of biological aging. Our study indicates that although the association of several inflammatory markers with rapid agers confirms their relationship with biological aging, they were not predictive enough to be included in the HAM index. On the other hand, our results suggest a potential interrelationship between senescence‐associated secretome, metabolism, and biological aging. Further studies would help us understand the mechanistic role of senescence in regulating age‐associated metabolites/metabolism.

Our study has several strengths, including a potential cohort design to identify metabolites related to biological age, covariable information, validation with an external cohort that used the same metabolomics platform, an approach to integrate metabolite and SASP/cytokine relationship, and causal inferencing to identify metabolites that theoretically are drivers of biological aging. However, it is also important to note the limitations of the current study. Owing to the limitations in the sample size and the availability of reference material, HAM index is developed from relative amounts of the metabolites rather than its absolute quantities. In addition, the impact of intestinal microbiome and dietary information can influence the analysis. Any unknown metabolites that were not included in the panel could benefit from the identification of chemical structures to provide new information. In the future, combining/comparing epigenetic clocks (Horvath, 2013; Liu et al., 2020; Simpson & Chandra, 2021) with our HAM index would be of great value to understand biological age and test whether they have a superior predictive capability in “younger” individuals. Furthermore, (1) the present study is based on a secondary analysis of frozen samples from a study designed to maximally identify genetic variations; (2) age and ageing are indeed confounded. However, this reverse nature of confounding may even strengthen our results as positive metabolites associated with healthy agers are despite the confounding chronological age. In addition, we correlated chronological age with the HAM index in the validation cohort, WRAP. Future studies to extend and optimize our integrated fingerprint to predict aging trajectories in humans as early as midlife are also needed. In conclusion, the present findings confirm that individuals showing signs of early or rapid aging have a distinct network of metabolites compared to healthy agers. Therefore, any therapeutic intervention to enhance the healthy lifespan of the elderly population should target a combination of metabolic processes rather than a single pathway.

## 4 MATERIALS AND METHODS

### 4.1 Participants

The Solve‐IT participants were recruited from several sources. Most were recruited through the University of Pittsburgh Claude D. Pepper Older Americans Independence Center, which maintains a registry of more than 2500 older adults who live in the greater Pittsburgh area and are interested in participating in clinical research. Print and radio ads were also used. Respondents were screened with a standardized phone interview. This study was approved by Institutional Review Board of University of Pittsburgh and complies with all relevant ethical regulations. An informed written consent was obtained from all participants. “Rapid” agers were age 65–75 years who could not walk up a flight of stairs or walk for 15 min without resting. “Healthy” agers were age 75 years and older who could walk up a flight of stairs or walk for 15 min without resting. We excluded participants with a history of a major cancer. Functional assessments on the cohort are further described in Breitbach et al. (2019).

The details of Wisconsin Registry of Alzheimer patients' (WRAP) cohort were described in the previous publications (Darst et al., 2019; Johnson et al., 2018). This registry measured a weaker walking ability than the one used for the SOLVE‐IT cohort. The WRAP data included information on whether the participant walks outside the house for 10 min without stopping. We used the following criteria to define healthy and rapid agers:

**Table:** 

Healthy agers		Rapid agers		Reference
Age	Walking outside for more than 10 min without stopping	Age	Walking outside for more than 10 min without stopping	
>70	More than once a week	>70	Rarely or never	Figure 3h
>65	More than once a week	>65	Rarely or never	Figure S3d
>60	More than once a week	>60	Rarely or never	Figure S3e
>55	More than once a week	>55	Rarely or never	Figure S3f
>50	More than once a week	>50	Rarely or never	Figure S3g
>45	More than once a week	>45	Rarely or never	Figure S3h

### 4.2 Cytokine and SASP analysis

SASP factors: CCL‐2/MCP‐1, IL1α, MMP‐1, PAI1, TNFα, IL‐6, and IL‐10 (Basisty et al., 2020; Camell et al., 2021; Englund et al., 2021; Huang et al., 2018). CCL‐2/MCP‐1, IL1α, MMP‐1, PAI1, and cystatin C were quantified using multiplex magnetic bead immunoassays (R&D Systems) based on Luminex xMAP multianalyte profiling platform (Luminex® 100/200™ System) according to the manufacturer's protocol and analyzed on Bio‐Rad Bio‐Plex Manager 6.1. Cytokine biomarkers analyzed included C‐reactive protein (CRP), tumor necrosis factor alpha (TNFα), and its receptors (TNFα‐R1 and TNFα‐R2), interleukin 6 soluble receptor (sIL‐6R), and interleukin 10 (IL‐10) as previously described (Langmann et al., 2017). For all proteins, more than 80% of the samples were within the detectable range. Undetectable targets were replaced with the lowest value for each protein.

### 4.3 Metabolomics

#### 4.3.1 Sample preparation

Samples were prepared by Metabolon, Inc. using the automated MicroLab STAR® system from Hamilton Company. Briefly, following addition of various internal standards, samples were deproteinated with methanol under vigorous shaking for 2 min, then centrifuged for 10 min at 680 *g*. The resultant extract was divided into five fractions, vacuum dried briefly to remove the organic solvents and stored under the nitrogen.

#### 4.3.2 Ultrahigh‐performance liquid chromatography–tandem mass spectroscopy (UPLC–MS/MS)

All platform data are LC–MS data (LC/MS Neg, LC/MS Pos Late, LC/MS Pos Early, and LC/MS Polar). Three independent reverse phase columns were used for the three RP/UPLC–MS/MS methods. This included two UPLC‐MS/MS methods with positive ion mode electrospray ionization (ESI), optimized for hydrophobic and hydrophilic compounds respectively, and one UPLC–MS/MS method with negative ion mode ESI. A HELIC column was used for the polar method (HILIC/UPLC–MS/MS with negative ion mode ESI). All methods utilized a Waters ACQUITY ultra‐performance liquid chromatography (UPLC) and a Thermo Scientific Q‐Exactive high‐resolution/accurate mass spectrometer interfaced with a heated electrospray ionization (HESI‐II) source and Orbitrap mass analyzer operated at 35,000 mass resolution. One aliquot was analyzed using acidic positive ion conditions, using C18 column (Waters UPLC BEH C18‐2.1×100 mm, 1.7 μm) with a gradient of water and methanol, containing 0.05% perfluoropentanoic acid (PFPA) and 0.1% formic acid (FA). Another aliquot was also analyzed using the same column but with a high organic gradient of methanol, acetonitrile, water, 0.05% PFPA, and 0.01% FA. Other two aliquots were analyzed using basic negative ion optimized conditions: (1) using C18 column (Waters UPLC BEH C18‐2.1×100 mm, 1.7 μm) eluted with gradient of methanol and water containing 6.5 mM ammonium bicarbonate at pH 8 2) using a HILIC column (Waters UPLC BEH Amide 2.1 × 150 mm, 1.7 μm) eluted with a gradient consisting of water and acetonitrile with 10 mM ammonium formate pH 10.8. Data‐dependent MS/MS analysis was performed with dynamic exclusion. The scan range varied slightly between methods but covered 70–1000 m/z. While some metabolites may be detected on more than one method, the final dataset is limited to identification on a single platform. This is guided by several criteria such as which platform yields the best signal to noise ratio.

#### 4.3.3 Data processing

Mass spec files were analyzed using Metabolon's inbuilt Laboratory Information Management System (LIMS). Compounds were identified by comparison to library entries of purified standards or recurrent unknown entities. Biochemical identifications were based on three criteria: retention index within a narrow RI window of the proposed identification, accurate mass match to the library ±10 ppm, and the MS/MS forward and reverse scores between the experimental data and authentic standards. Peaks were quantified using area‐under‐the‐curve and corrected for batch variations and normalized to the median value. Median scaling is less prone to skewing than normalizing to means. Median scaling is also performed to correct for batch to batch variability. Essentially, each compound is corrected in run‐day blocks by registering the medians to equal one (1.00) and normalizing each data point proportionately. The missing values were replaced with the lowest value for each sample. Overall, about 15% datapoints were missing from the original data.

### 4.4 Statistical analysis

#### 4.4.1 Correlation matrix and chord diagram

We computed Spearman's rank correlation coefficients (*ρ*), and false discovery rate (FDR) method to correct *p*‐values for multiplicity. A threshold of corrected *p <* 0.05 was used to identify correlations between super pathways. R functions rcorr in package HMISC, p.adjust, and chordDiagram in circularize were used for analysis.

#### 4.4.2 Orthogonal projection of partial least square discriminant analysis (OPLS‐DA)

OPLS‐DA method was used to test whether there is any differences between the metabolome of the healthy agers and rapid agers. Being a supervised dimension reduction analysis OPLS‐DA enable the reduction of complexity of the data and recognize the patterns, trends and interactions between metabolites. Data were classified into rapid and healthy agers as described (Breitbach et al., 2019) and OPLS‐DA modeling included sevenfold cross validation with 200 iteration in each step. Both predictive and orthogonal scores of the best model were extracted and plotted. Metabolites with variable importance of the Projection (VIP) score more than 1 was used in subsequent analyses such as the model prediction. For rapid and healthy agers, we used the dataset excluding unknown metabolites whereas in other analysis, the entire set of 1327 metabolites were used. Sartorius SIMCA 16.0 software (Sartorius stedim biotech, Goettingen, Germany) was used for analysis.

Receiver operator characteristic (ROC) curve analysis was performed using roc function in pROC package. We used healthy and rapid agers as the dichotomous dependent variable. Excess of area under the curve (AUC) over 0.5 was interpreted as representing the strength of the association, 95% confidence bands for AUC were constructed, and bootstrap methods were used for comparison of AUCs. R packages ggplot2 and roc function in fbroc, and roc.test function in pROC package were used for analysis.

#### 4.4.3 Calculation of AUC_comb_ values

Receiver operating characteristic (ROC) curve analysis is a robust classification tool used to determine if an individual metabolite can distinguish between groups while accounting for confounding factors. We first defined healthy agers as cases and rapid agers as controls and calculated the ROC_AUC values (ROC_AUC_HA_), then the controls and cases were reversed and the ROC_AUC values for predicting rapid agers (ROC_AUC_RA_) was calculated. Since ROC_AUC_HA_ = 1‐ROC_AUC_RA_,we combined these values into one variable named AUC_comb_ as follows:

$${\mathrm{AUC}}_{\text{comb}}=\left\{\begin{array}{ccc} \mathrm{ROC}\_{\mathrm{AUC}}_{\mathrm{HA}} & \text{if}, & \mathrm{ROC}\_{\mathrm{AUC}}_{\mathrm{HA}}>0.5\\ 1-\left(\mathrm{ROC}\_{\mathrm{AUC}}_{\mathrm{RA}}\;\right) & \text{if}, & \mathrm{ROC}\_{\mathrm{AUC}}_{\mathrm{RA}}>0.5\\ \mathrm{ROC}\_{\mathrm{AUC}}_{\mathrm{HA}}\;\text{or}\;\mathrm{ROC}\_A{\mathrm{UC}}_{\mathrm{RA}} & \text{if}, & \mathrm{ROC}\_{\mathrm{AUC}}_{\mathrm{HA}}=\mathrm{ROC}\_{\mathrm{AUC}}_{\mathrm{RA}}=0.5 \end{array}\right.$$

As a result, AUC_comb_ > 0.5 will denote greater predictive power of a metabolite toward heathy agers and AUC_comb_ < 0.5 will denote greater predictive power toward rapid agers and AUC_comb_ = 0.5 indicates no predictive capacity.

#### 4.4.4 Statistical modeling of healthy aging

To streamline the selection of metabolites while minimizing the inclusion of confounding variables that might be stronger predictors of healthy and rapid aging, we employed a linear regression model. Linear regression models are valuable statistical tools for deriving straightforward and quantifiable models. 253 known metabolites with VIP >1 in separating healthy from rapid agers were selected for building a mathematical model of healthy aging. In order to identify a more easily interpretable and a relatively less correlated subset of metabolites we used LASSO linear regression for developing the model. The *λ* parameter that results in the lowest cross validation error following 10‐fold cross validation was chosen to build the model. We used 1000 internal bootstrapping replicates with five external bootstrapping cycles. The final model consisted of 25 metabolites including, 1‐stearoyl‐2‐docosahexaenoyl‐GPE (18:0/22:6), 3,4‐dihydroxybutyrate, 3‐hydroxyhexanoylcarnitine, 5‐hydroxylysine, 5‐oxoproline, beta‐cryptoxanthin, citrate, cortisone, eicosenoylcarnitine (C20:1), glucuronate, isocitrate, isoleucine, N6,N6,N6‐trimethyllysine, N‐acetylaspartate (NAA), N‐acetyl‐beta‐alanine, pimelate (C7‐DC), prolylhydroxyproline, and vanillylmandelate (VMA). Using the the coefficients form the model we define the Healthy Aging Metabolic index (HAMI) as $\text{HAMI}=\sum_{i=1}^{25}a_{i}m_{i}$, where $a_{1}\ldots a_{25}$ are the LASSO coefficients, and $m\ldots m_{25}$ are the concentrations of metabolites as shown in Figure 3e. R packages elasso and glmnet were used for analysis.

#### 4.4.5 Pathway enrichment analysis

The PubChem ID of significant metabolites and the *p*‐values were used as the input for the ingenuity pathway analysis (IPA). Pathway‐enrichment analysis was performed using Qiagen Ingenuity Pathway Analysis (Qiagen, Redwood city, CA). IPA and interpretation were based on the comprehensive and manually curated content of the Ingenuity Knowledge Base, which organizes biological interactions and functional annotations created from primary literature and public and third‐party databases.

#### 4.4.6 Causal discovery computation

The causal network was computed using a R script that used the function called pcstable from the R package bnlearn. The alpha value was set at 0.05 and 100 bootstrap iterations were executed.

## AUTHOR CONTRIBUTIONS

AUG, SLG, NR, and SP provided study design. SLG, NR, and SP assembled study cohort. SH, TA, SP, GN, and AUG acquired, analyzed, or interpreted data. DP and AL helped with running Luminex assays. SP and GN provided feedback on statistical analyses. SO and GN performed causal inferencing studies. SH, TA, and AUG drafted the manuscript with the help of all coauthors. AUG supervised the study.

## CONFLICT OF INTEREST STATEMENT

None declared.

## CODE AVAILABILITY

Details of R functions and packages used in this study were described in methods section.

## Supporting information


Appendix S1

## Data Availability

The source data underlying Figures 2b, 4c,d, 5e, 6e, 7e are provided within the supplied Source Data file. Data are available at: https://www.ebi.ac.uk/metabolights/editor/MTBLS1991/descriptors.
