# Supplementary material for: A molecular index for biological age identified from the metabolome and senescence‐associated secretome in humans
Source: Aging Cell. 2024 Mar 7;23(4):e14104. doi: 10.1111/acel.14104 (PMC11019119; doi:10.1111/acel.14104)
Supplement: Supplementary file 1 — Appendix S1 [file ACEL-23-e14104-s001.pdf]

Figure. S1

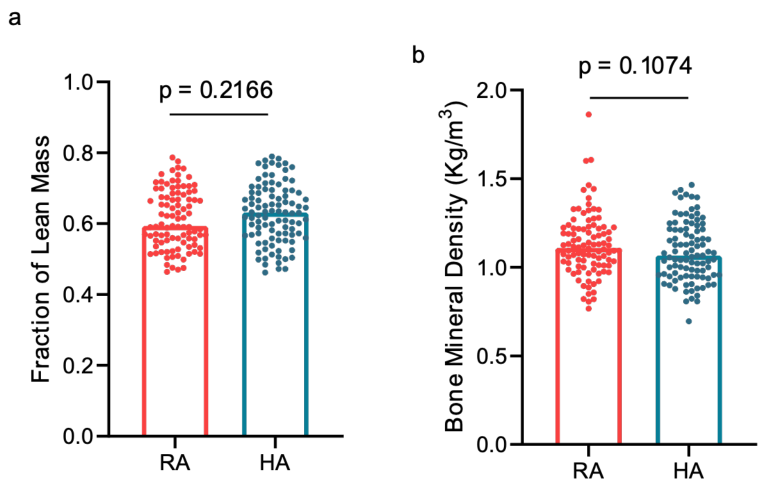

Supplementary figure 1: Distribution of fraction of a) body lean mass and b) bone mineral density in rapid agers and healthy agers.

Figure. S2

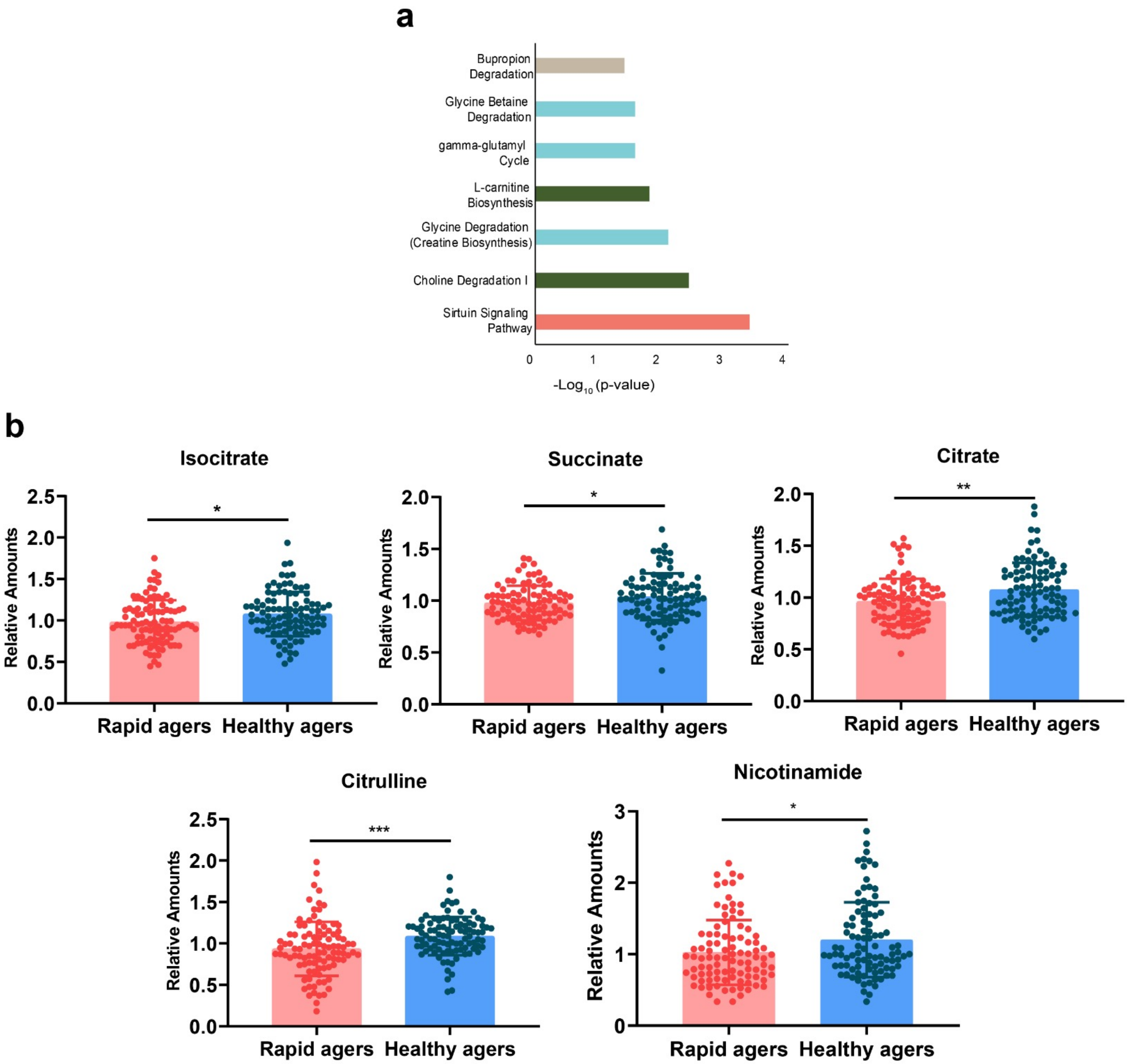

Supplementary Figure 2: a. Pathways associated with healthy agers **b.** Distribution of metabolites involved in sirtuin signaling pathway among rapid (RA) and healthy (HA) agers. *, $p<0.05$, **, $p<0.01$, ***, $p<0.001$, Student's-t test

Figure. S3

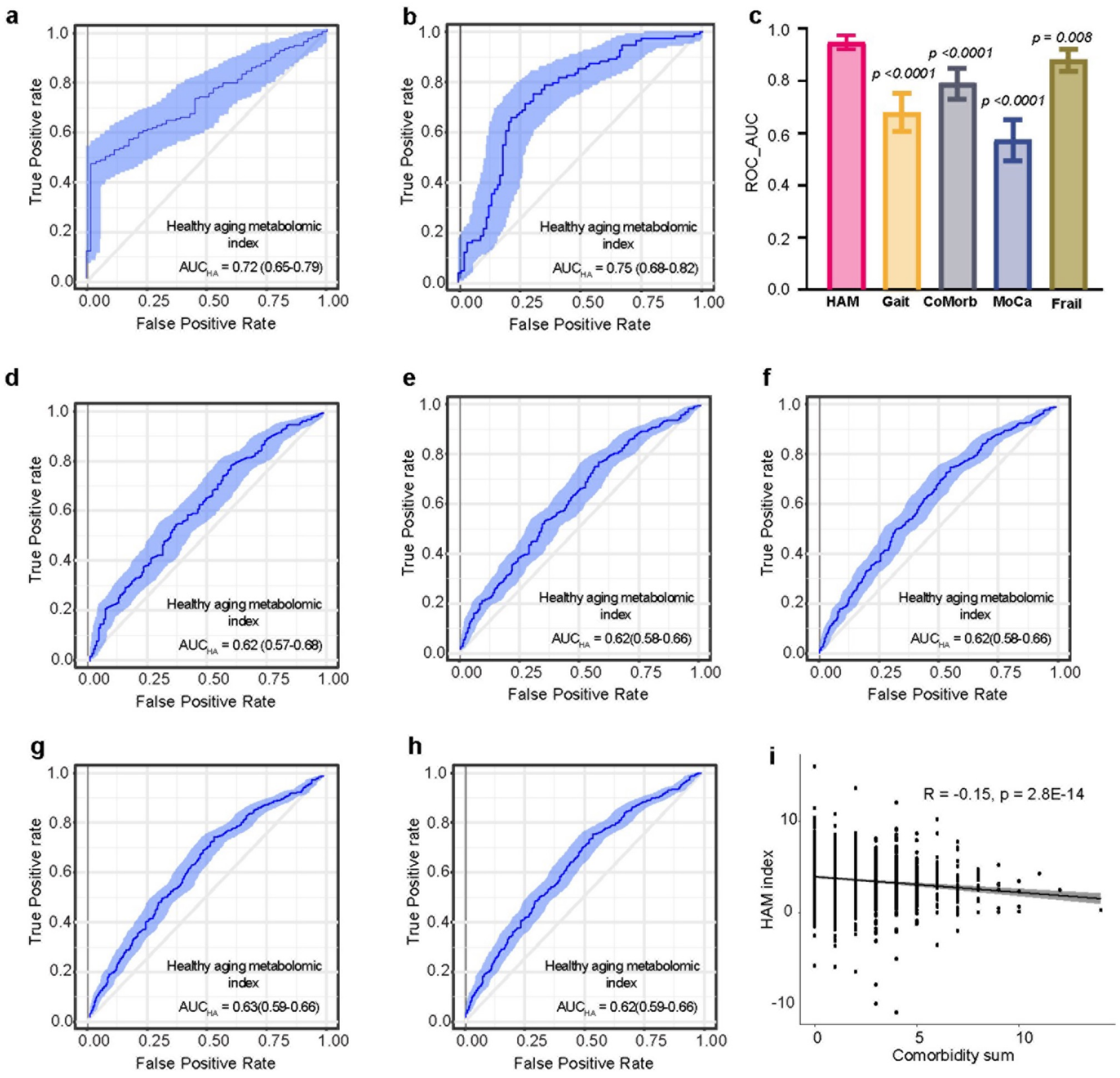

Supplementary Figure 3. a) AUC value of HAM index in differentiating seniors based on b) frailty (group 0,1 vs group 2) c) comorbidity (less than 4 vs equal and greater than 4). c) The AUC value of HAM index is significantly higher than other physiological aging indices. p-value vs. HAM index between AUC values. ROC curves comparing the ability of the HAM index to distinguish between individuals over the age of d) 65, e) 60, f) 55, g) 50, and h) 45 who are capable of walking for more than 10 minutes at least once per week versus those who rarely walk for more than 10 minutes in the WRAP cohort. i) HAM index in the test cohort showed a significant correlation with the comorbidities.

Figure. S4

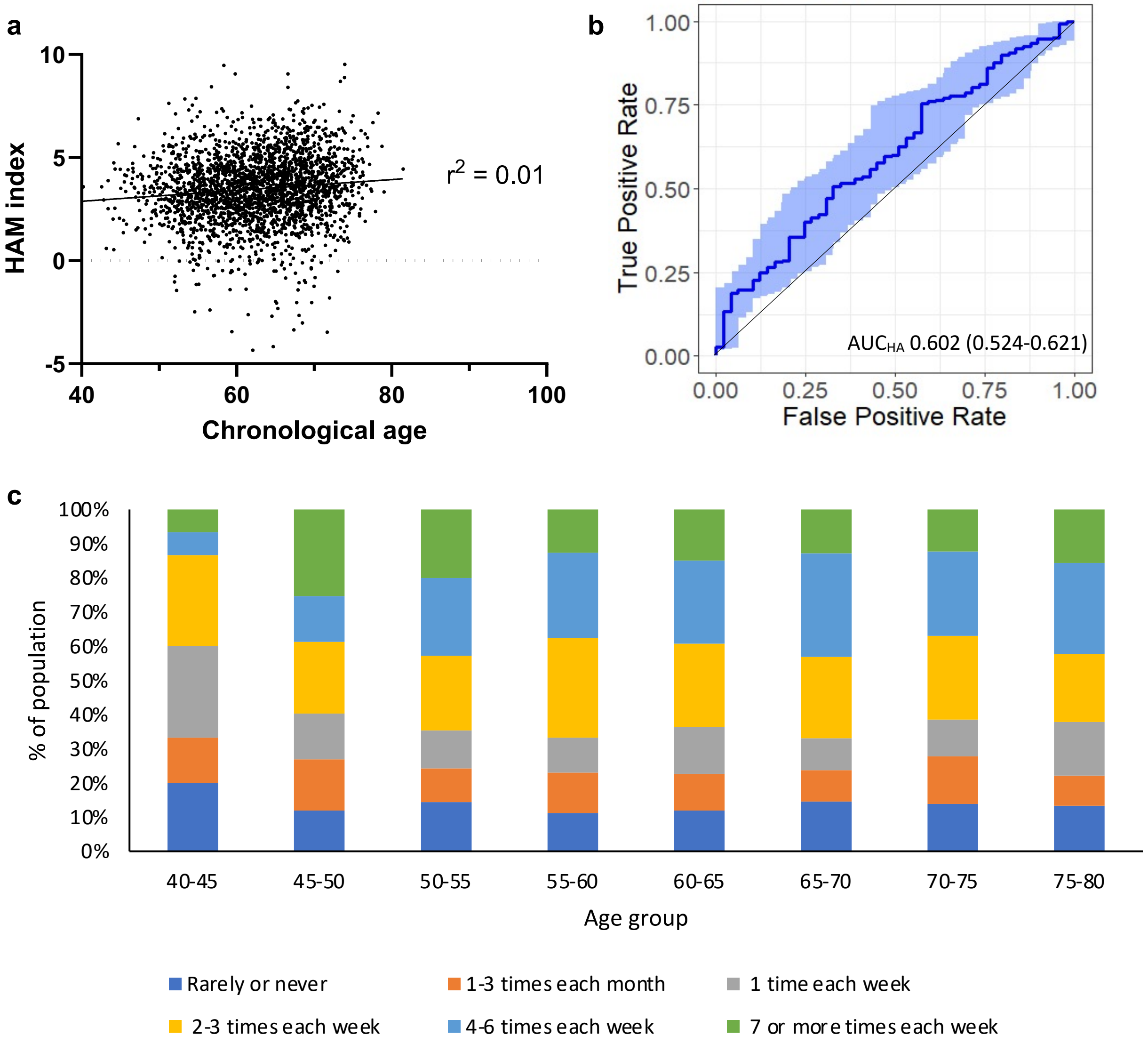

Supplementary Figure 4. a) Dot plot showing the distribution of HAM index across age in WRAP data b) ROC curve for ham index in differentiating over 70 who rarely walk outside, and people aged 60-70 who walk at least once a week. c) Across all age groups, approximately 10% of participants reported rarely or never walking outside for more than 10 minutes. Conversely, approximately 65% of the population in all age groups reported engaging in outdoor walks at least weekly.

Figure. S5

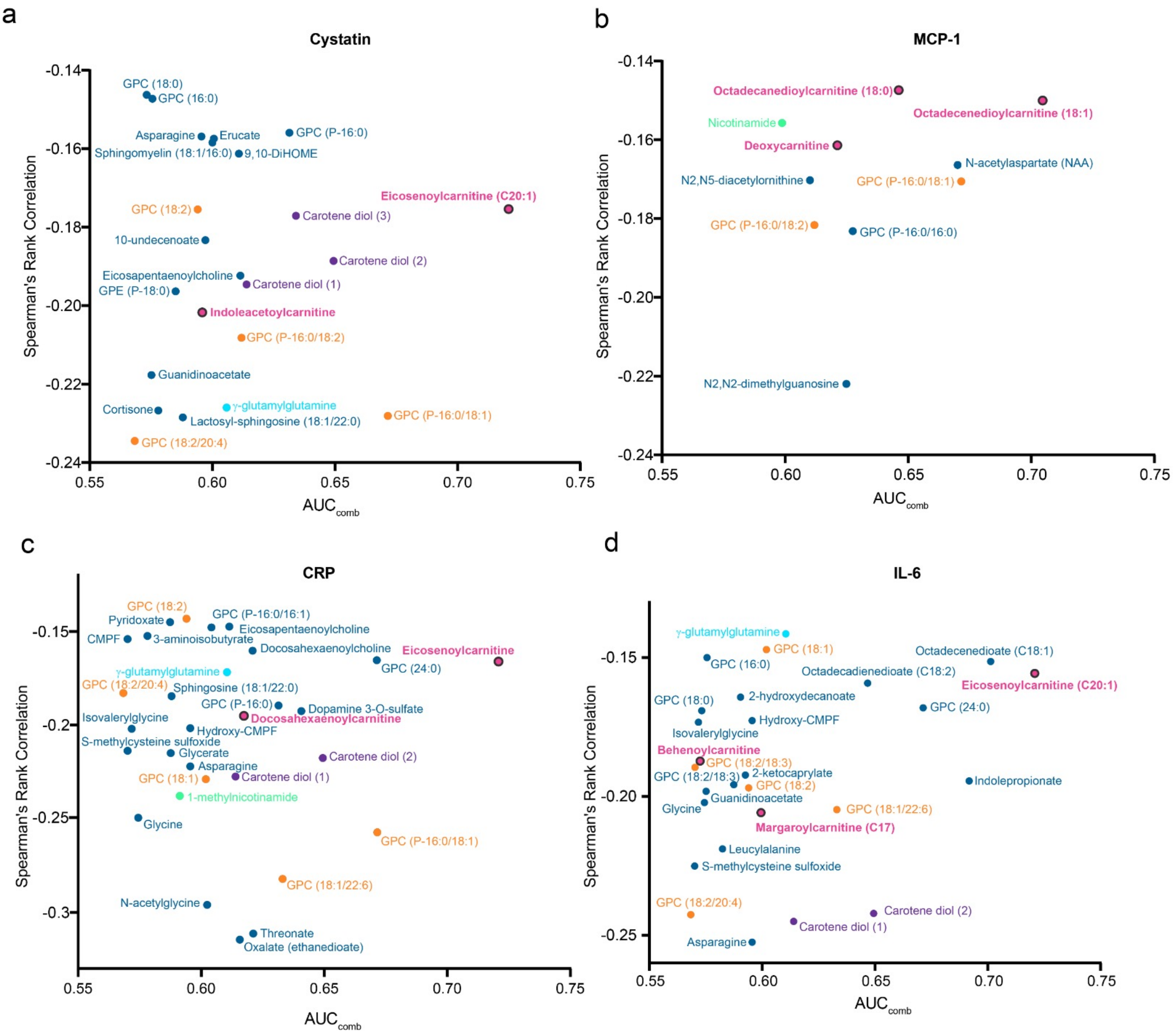

Supplementary Figure 5. Dot plot showing spearman's rank correlation between SASP or proinflammatory markers and the metabolites vs. AUC_{comb} values of the metabolites. The metabolites that are positively associated with healthy agers and negatively associated with circulating factors were analyzed. Only significant endogenous metabolites were plotted ($p< 0.05$). Common metabolites were represented in pink (acylcarnitine), orange (oleic or linoleic acid containing glycerol phosphatidylcholine), purple(carotene diol), cyan (γ-glutamylgutamate) and green (nicotinamide).

Figure. S6

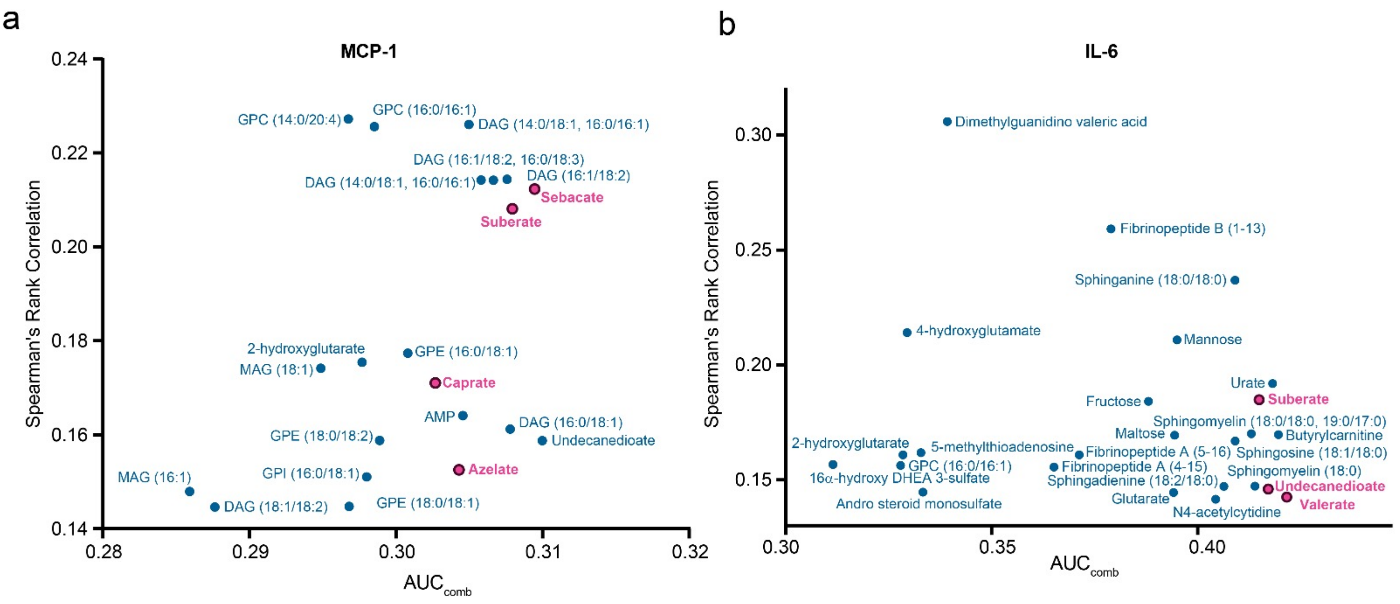

Supplementary Figure 6. Dot plot showing spearman's rank correlation between SASP markers, MCP-1 (a), IL-6 (b), and the metabolites vs. AUCcomb values of the metabolites. Correlations were calculated only for the metabolites that are, positively associated with healthy agers as well as negatively associated with the SASP factors. Significant endogenous metabolites were plotted ($p < 0.05$). Dicarboxylic acids (pink) were the common metabolites associated with both MCP-1 and IL-6.

Figure. S7

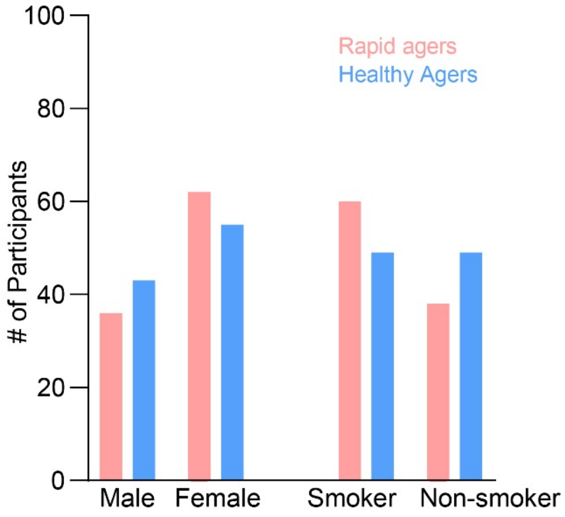

Supplementary Figure 7. Column chart showing number of healthy agers and rapid agers in each category of the study population.

Figure. S8

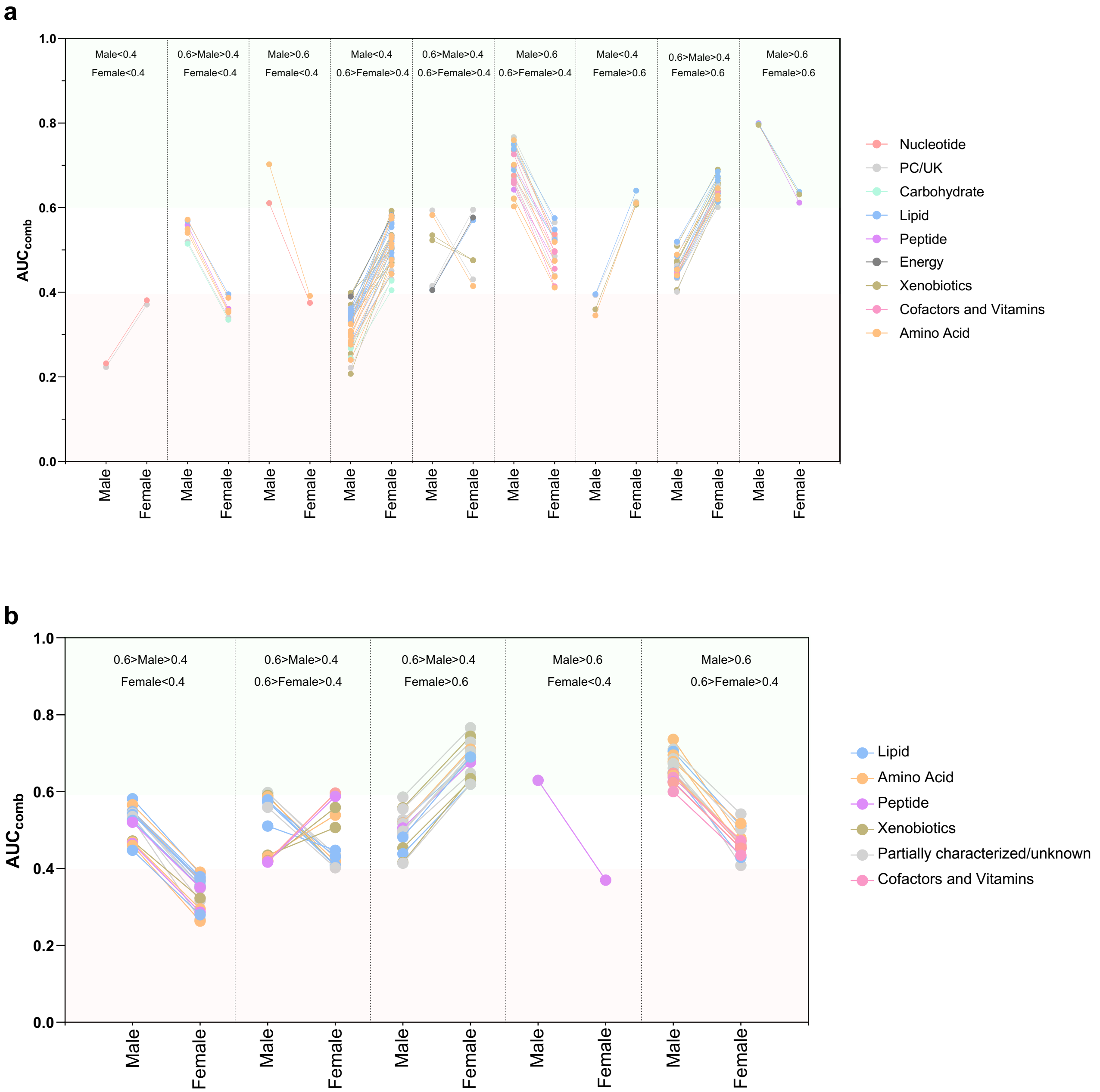

Supplementary Figure 8. a) Interaction plot showing the AUC_{comb} of metabolites that significantly differ between male and female groups in predicting healthy agers. AUC_{comb} > 0.5 values = healthy agers, AUC_{comb} <0.5 values = rapid agers. Metabolites are color coded based on their super pathways b) Differences in AUC of metabolites among the smoking and non-smoking population and its influence on predicting healthy aging is shown as an interaction plot.

Figure. S9

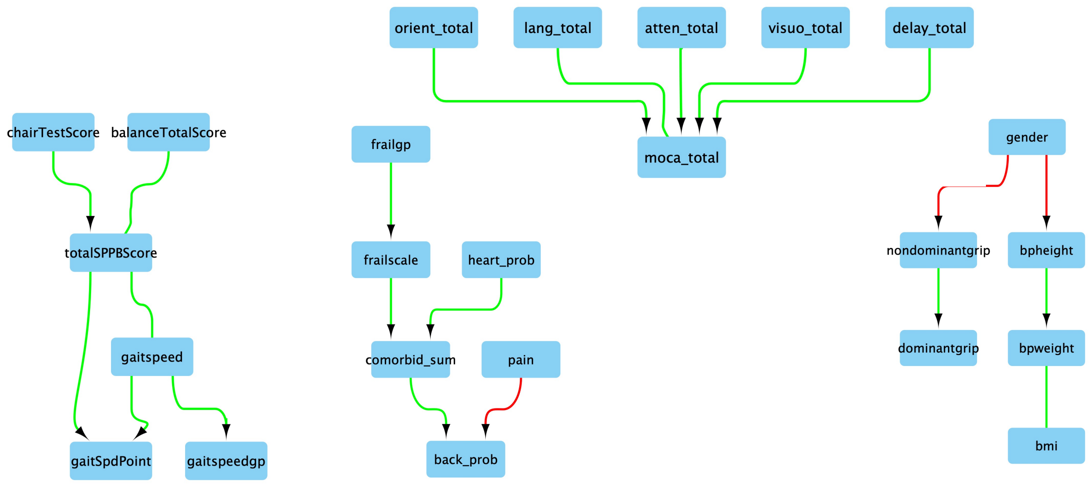

Supplementary Figure 9. Causal network shows inferred relationships between several clinical features in the SOLVE-IT cohort.

Figure. S10

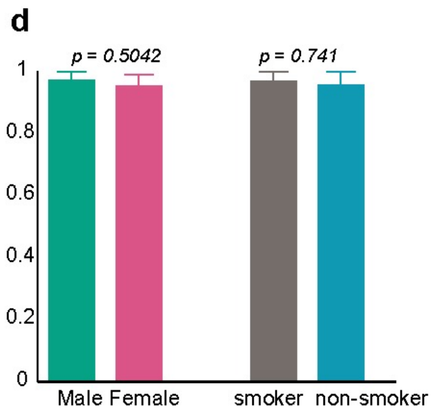

Supplementary Figure 10. Column chart showing the ROC-AUCHA values of HAM in predicting healthy agers among categories in gender and smoking status. p values were calculated between each pair.

	HAM vs. gaitSpdPoint	HAM vs. balanceTotalScore	HAM vs. chairTestScore	HAM vs. totalSPPB Score	HAM vs. gaitspeed	HAM vs. comorbid_sum	HAM vs. dominant grip	HAM vs. nondominantgrip	HAM vs. digcor	HAM vs. visuo_total	HAM vs. naming_total	HAM vs. atten_total	HAM vs. lang_total	HAM vs. abstr_total	HAM vs. delay_total	HAM vs. orient_total	HAM vs. moca_total
Spearman r																	
r	0.07457	0.0704	0.1166	0.1172	0.1423	-0.4224	0.04402	0.06217	-0.1067	-0.01647	-0.05483	0.0565	-0.08269	0.03887	-0.1048	-0.09334	-0.07765
95% confidence interval	-0.07043 to 0.2	-0.07460 to 0.21	-0.02811 t	-0.02750 t	-0.002324	-0.5341 to	-0.1009 to	-0.08318 t	-0.2471 to	-0.1603 to	-0.1975 to	-0.08846 t	-0.2243 to 0.06	-0.1060 to 0.1	-0.2453 to 0.0400	-0.2344 to 0.05159	-0.2194 to 0.06735
P value																	
P (two-tailed)	0.2989	0.3269	0.1036	0.1018	0.0472	<0.0001	0.5401	0.3879	0.1368	0.8187	0.4453	0.4316	0.2492	0.5886	0.1438	0.1932	0.2794
P value summary	ns	ns	ns	ns	*	****	ns	ns	ns	ns	ns	ns	ns	ns	ns	ns	ns
Exact or approximate P value?	Approximate	Approximate	Approxima	Approxima	Approxima	Approxima	Approxima	Approxima	Approxima	Approxima	Approxima	Approxima	Approximate	Approximate	Approximate	Approximate	Approximate
Significant? (alpha = 0.05)	No	No	No	No	Yes	Yes	No	No	No	No	No	No	No	No	No	No	No
Number of XY Pairs	196	196	196	196	195	196	196	195	196	196	196	196	196	196	196	196	196

	HAM vs. pfi	HAM vs. rolp	HAM vs. role	HAM vs. soc	HAM vs. pain	HAM vs. mhi	HAM vs. vital	HAM vs. ghp	HAM vs. frailscale
Spearman r									
r	0.4368	0.3281	0.2435	0.3001	0.4055	0.1879	0.3085	0.399	-0.3788
95% confidence interval	0.3122 to 0.546	0.1930 to 0.451	0.1029 to	0.1629 to	0.2775 to	0.04486 to	0.1719 to	0.2703 to	-0.4960 to -0.2481
P value									
P (two-tailed)	<0.0001	<0.0001	0.0006	<0.0001	<0.0001	0.0084	<0.0001	<0.0001	<0.0001
P value summary	****	****	***	****	****	**	****	****	****
Exact or approximate P value?	Approximate	Approximate	Approxima	Approxima	Approxima	Approxima	Approxima	Approxima	Approximate
Significant? (alpha = 0.05)	Yes	Yes	Yes	Yes	Yes	Yes	Yes	Yes	Yes
Number of XY Pairs	196	196	196	196	196	196	196	196	196

gaitSpdPoint = Gait Speed Point
HAM = Healthy Aging Metabolic Index
SPPB = Short Physical Performance Battery
comorbid_sum = co-morbidities (total)
lang = language
abstr = abstract
moca = Montreal Cognitive Assessment
Digcor = Digit Symbol Substitution Test Score
visuo_total = Montreal Cognitive Assessment (MoCA) visuospatial/executive subscore
pfi= Short Form-36 (SF-36) Physical Function Index
rolp=SF-36 Role-Physical score
role=SF-36 Role-Emotional score
soc=SF-36 Social Function Index
mhi=SF-36 Mental Health Index
ghp=SF-36 General Health Perceptions score

Supplementary table 2: Senesence associated SASP and cytokines measured in this study.

IL-1	(Interleukin 1α) Cytokine responsible for production of inflammation	[3], [4]
MMP-1	(Matrix metalloproteinase-1) interstitial and fibroblast collagenase	[7], [8]
CRP-1	C-reactive protein	[11,12]
TNFα-R1 and TNFα-R2	Receptors of Tumor necrosis factor alpha	[11,14]
sIL-6R	Soluble receptor of Interleukin-6	[11,16]

References

1. Conley, M. N. et al. Aging and serum MCP-1 are associated with gut microbiome composition in a murine model. PeerJ 4, e1854, doi:10.7717/peerj.1854 (2016).
2. Chiao, Y. A. et al. Multi-analyte profiling reveals matrix metalloproteinase-9 and monocyte chemotactic protein-1 as plasma biomarkers of cardiac aging. Circulation. Cardiovascular genetics 4, 455-462, doi:10.1161/circgenetics.111.959981 (2011).
3. Orjalo, A. V., Bhaumik, D., Gengler, B. K., Scott, G. K. & Campisi, J. Cell surface-bound IL-1alpha is an upstream regulator of the senescence-associated IL-6/IL-8 cytokine network. Proceedings of the National Academy of Sciences of the United States of America 106, 17031-17036, doi:10.1073/pnas.0905299106 (2009).
4. Mariotti, M., Castiglioni, S., Bernardini, D. & Maier, J. A. Interleukin 1 alpha is a marker of endothelial cellular senescent. Immunity & ageing : I & A 3, 4, doi:10.1186/1742-4933-3-4 (2006).
5. Mathews, P. M. & Levy, E. Cystatin C in aging and in Alzheimer's disease. Ageing Res Rev 32, 38-50, doi:10.1016/j.arr.2016.06.003 (2016).
6. Shlipak, M. G. et al. Cystatin C and mortality risk in the elderly: the health, aging, and body composition study. J Am Soc Nephrol 17, 254-261, doi:10.1681/asn.2005050545 (2006).

7. Noh, E. M. et al. PTEN inhibits replicative senescence-induced MMP-1 expression by regulating NOX4-mediated ROS in human dermal fibroblasts. Journal of cellular and molecular medicine 21, 3113-3116, doi:10.1111/jcmm.13220 (2017).
8. Vierkötter, A. et al. MMP-1 and -3 promoter variants are indicative of a common susceptibility for skin and lung aging: results from a cohort of elderly women (SALIA). The Journal of investigative dermatology 135, 1268-1274, doi:10.1038/jid.2015.7 (2015).
9. Kortlever, R. M., Higgins, P. J. & Bernards, R. Plasminogen activator inhibitor-1 is a critical downstream target of p53 in the induction of replicative senescence. Nature cell biology 8, 877-884, doi:10.1038/ncb1448 (2006).
10. Khan, S. S. et al. A null mutation in SERPINE1 protects against biological aging in humans. Science advances 3, eaao1617, doi:10.1126/sciadv.aao1617 (2017).
11. Langmann, G. A. et al. Inflammatory Markers and Frailty in Long-Term Care Residents. Journal of the American Geriatrics Society 65, 1777-1783, doi:10.1111/jgs.14876 (2017).
12. Stojanović, S. D., Fiedler, J., Bauersachs, J., Thum, T. & Sedding, D. G. Senescence-induced inflammation: an important player and key therapeutic target in atherosclerosis. European heart journal, doi:10.1093/eurheartj/ehz919 (2020).
13. Bruunsgaard, H., Andersen-Ranberg, K., Hjelmborg, J., Pedersen, B. K. & Jeune, B. Elevated levels of tumor necrosis factor alpha and mortality in centenarians. The American journal of medicine 115, 278-283, doi:10.1016/s0002-9343(03)00329-2 (2003).
14. Marti, C. N. et al. Soluble tumor necrosis factor receptors and heart failure risk in older adults: Health, Aging, and Body Composition (Health ABC) Study. Circulation. Heart failure 7, 5-11, doi:10.1161/circheartfailure.113.000344 (2014).
15. Harris, T. B. et al. Associations of elevated interleukin-6 and C-reactive protein levels with mortality in the elderly. The American journal of medicine 106, 506-512, doi:10.1016/s0002-9343(99)00066-2 (1999).
16. Giuliani, N. et al. Serum interleukin-6, soluble interleukin-6 receptor and soluble gp130 exhibit different patterns of age- and menopause-related changes. Exp Gerontol 36, 547-557, doi:10.1016/s0531-5565(00)00220-5 (2001).
17. Welsh, P. et al. Circulating interleukin-10 and risk of cardiovascular events: a prospective study in the elderly at risk. Arteriosclerosis, thrombosis, and vascular biology 31, 2338-2344, doi:10.1161/atvbaha.111.231795 (2011).

Supplementary Table 3: Spearman's Rank Correlation of Metabolites with C-Reactive Protein (CRP)

BIOCHEMICAL	Amino Acids	Sub Pathway	Estimate	*p*-value	Statistics	FDR
cortolone glucuronide (1)	Lipids	Corticosteroids	0.31476	7.83E-06	4.59499	0.00234
Fibrinopeptide A (5-16)*	Carbohydrates	Fibrinogen Cleavage Peptide	0.31365	8.45E-06	4.57708	0.00234
ADSGEGDFXAEGGGVR*	Cofactors and Vitamins	Fibrinogen Cleavage Peptide	0.31042	1.06E-05	4.52487	0.00234
Fibrinopeptide A (2-15)	Peptides	Fibrinogen Cleavage Peptide	0.30568	1.46E-05	4.44853	0.00277
Urate	Nucleotides	Purine Metabolism, (Hypo)Xanthine/Inosine containing	0.29552	2.87E-05	4.28621	0.00423
Fibrinopeptide B (1-13)	Peptide	Fibrinogen Cleavage Peptide	0.29312	3.36E-05	4.2482	0.00436
Mannose	Carbohydrate	Fructose, Mannose and Galactose Metabolism	0.29194	3.62E-05	4.22944	0.00436
andro steroid monosulfate C19H28O6S (1)*	Lipid	Androgenic Steroids	0.27643	9.55E-05	3.98568	0.00913
dimethylguanidino valeric acid (DMGV)*	Amino Acid	Urea cycle; Arginine and Proline Metabolism	0.27628	9.64E-05	3.98321	0.00913
16a-hydroxy DHEA 3-sulfate	Lipid	Androgenic Steroids	0.26557	0.00018	3.81694	0.01401
Fibrinopeptide A (3-15)	Peptide	Fibrinogen Cleavage Peptide	0.26492	0.00019	3.80685	0.01401
N-acetylphenylalanine	Amino Acid	Phenylalanine Metabolism	0.24814	0.00049	3.54939	0.02926

BIOCHEMICAL	Amino Acids	Sub Pathway	Estimate	*p*-value	Statistics	FDR
Fibrinopeptide A (4-15)	Peptide	Fibrinogen Cleavage Peptide	0.23044	0.00123	3.28134	0.05085
DSGEGDFXAEGGGVR*	Peptide	Fibrinogen Cleavage Peptide	0.22846	0.00136	3.25167	0.05285
pregnenetriol sulfate*	Lipid	Pregnenolone Steroids	0.22509	0.0016	3.20105	0.05828
4-hydroxyglutamate	Amino Acid	Glutamate Metabolism	0.22425	0.00167	3.18853	0.05828
aspartate	Amino Acid	Alanine and Aspartate Metabolism	0.22425	0.00167	3.1885	0.05828
N-acetylkynurenine (2)	Amino Acid	Tryptophan Metabolism	0.22181	0.00188	3.152	0.06086
androstenediol (3beta,17beta) disulfate (2)	Lipid	Androgenic Steroids	0.21221	0.00297	3.00906	0.07639
Fibrinopeptide A (3-16)	Peptide	Fibrinogen Cleavage Peptide	0.2122	0.00297	3.00886	0.07639
21-hydroxypregnenolone disulfate	Lipid	Pregnenolone Steroids	0.21205	0.003	3.00656	0.07639
kynurenine	Amino Acid	Tryptophan Metabolism	0.20909	0.00344	2.9627	0.08594
3-(4-hydroxyphenyl)lactate (HPLA)	Amino Acid	Tyrosine Metabolism	0.205	0.00414	2.90225	0.09977
arachidonoyl ethanolamide	Lipid	Endocannabinoid	0.20096	0.00496	2.84257	0.10577
bradykinin, des-arg(9)	Peptide	Polypeptide	0.19906	0.00539	2.81451	0.10577
hydantoin-5-propionate	Amino Acid	Histidine Metabolism	0.19807	0.00563	2.80005	0.10823
5-(galactosylhydroxy)-L-lysine	Amino Acid	Lysine Metabolism	0.19494	0.00645	2.75402	0.11754

**BIOCHEMICAL**	**Amino Acids**	**Sub Pathway**	**Estimate**	***p*-value**	**Statistics**	**FDR**
cysteine sulfinic acid	Amino Acid	Methionine, Cysteine, SAM and Taurine Metabolism	0.19481	0.00649	2.75204	0.11754
androstenediol (3beta,17beta) monosulfate (2)	Lipid	Androgenic Steroids	0.1945	0.00658	2.74754	0.11754
palmitoyl-palmitoyl-glycerol (16:0/16:0) [2]*	Lipid	Diacylglycerol	0.19258	0.00714	2.71939	0.12138
4-hydroxyphenylacetylglutamine	Peptide	Acetylated Peptides	0.19098	0.00764	2.69589	0.12513
N-acetyltryptophan	Amino Acid	Tryptophan Metabolism	0.18604	0.0094	2.62365	0.1438
N-acetylcarnosine	Amino Acid	Histidine Metabolism	0.18595	0.00943	2.62234	0.1438
11beta-hydroxyandrosterone glucuronide	Lipid	Androgenic Steroids	0.18547	0.00963	2.61524	0.1438
sucrose	Carbohydrate	Disaccharides and Oligosaccharides	0.18514	0.00975	2.61051	0.1438
maltose	Carbohydrate	Glycogen Metabolism	0.18438	0.01006	2.5994	0.1438
beta-citrylglutamate	Amino Acid	Glutamate Metabolism	0.18327	0.01053	2.58319	0.1438
N-acetylthreonine	Amino Acid	Glycine, Serine and Threonine Metabolism	0.18269	0.01078	2.57481	0.1438
pregnenetriol disulfate*	Lipid	Pregnenolone Steroids	0.18258	0.01083	2.5732	0.1438
gamma-CEHC glucuronide*	Cofactors and Vitamins	Tocopherol Metabolism	0.18161	0.01127	2.55908	0.1438
glutamate	Amino Acid	Glutamate Metabolism	0.18059	0.01174	2.54417	0.14415
gamma-CEHC	Cofactors and Vitamins	Tocopherol Metabolism	0.1802	0.01193	2.53847	0.1451

BIOCHEMICAL	Amino Acids	Sub Pathway	Estimate	*p*-value	Statistics	FDR
N-acetyltyrosine	Amino Acid	Tyrosine Metabolism	0.17911	0.01246	2.52254	0.1463
palmitoyl-oleoyl-glycerol (16:0/18:1) [1]*	Lipid	Diacylglycerol	0.17888	0.01258	2.51921	0.1463
3-methoxytyrosine	Amino Acid	Tyrosine Metabolism	0.1774	0.01334	2.49775	0.15355
N-acetylmethionine	Amino Acid	Methionine, Cysteine, SAM and Taurine Metabolism	0.17707	0.01352	2.49287	0.15355
androstenediol (3alpha, 17alpha) monosulfate (2)	Lipid	Androgenic Steroids	0.17586	0.01418	2.47532	0.15799
androstenediol (3beta,17beta) disulfate (1)	Lipid	Androgenic Steroids	0.17547	0.01439	2.46974	0.15905
delta-CEHC	Cofactors and Vitamins	Tocopherol Metabolism	0.17423	0.01511	2.4517	0.16426
1-dihomo-linolenylglycerol (20:3)	Lipid	Monoacylglycerol	0.1726	0.0161	2.42805	0.16681
alpha-hydroxyisovalerate	Amino Acid	Leucine, Isoleucine and Valine Metabolism	0.17134	0.01691	2.4098	0.17112
delta-CEHC glucuronide*	Cofactors and Vitamins	Tocopherol Metabolism	0.17038	0.01754	2.39588	0.17398
alpha-hydroxyisocaproate	Amino Acid	Leucine, Isoleucine and Valine Metabolism	0.16858	0.01879	2.36988	0.17666
2-methylbutyrylcarnitine (C5)	Amino Acid	Leucine, Isoleucine and Valine Metabolism	0.16784	0.01932	2.35915	0.18042
Fibrinopeptide B (1-11)	Peptide	Fibrinogen Cleavage Peptide	0.16516	0.02137	2.32043	0.19121
5-methylthioadenosine (MTA)	Amino Acid	Polyamine Metabolism	0.1645	0.0219	2.31087	0.19234

BIOCHEMICAL	Amino Acids	Sub Pathway	Estimate	*p*-value	Statistics	FDR
glycerophosphoserine*	Lipid	Phospholipid Metabolism	0.16378	0.02249	2.30053	0.19494
indole-3-carboxylate	Amino Acid	Tryptophan Metabolism	0.16301	0.02314	2.2894	0.19928
glycosyl-N-stearoyl-sphingosine (d18:1/18:0)	Lipid	Hexosylceramides (HCER)	-0.1641	0.02225	-2.3048	0.1941
12,13-DiHOME	Lipid	Fatty Acid, Dihydroxy	-0.1646	0.02184	-2.312	0.19234
ceramide (d16:1/24:1, d18:1/22:1)*	Lipid	Ceramides	-0.165	0.02149	-2.3183	0.19121
1-lignoceroyl-GPC (24:0)	Lipid	Lysophospholipid	-0.1655	0.02109	-2.3254	0.19028
eicosenoylcarnitine (C20:1)*	Lipid	Fatty Acid Metabolism (Acyl Carnitine, Monounsaturated)	-0.1662	0.02053	-2.3359	0.18646
sphingomyelin (d18:1/20:0, d16:1/22:0)*	Lipid	Sphingomyelins	-0.1665	0.02035	-2.3393	0.18608
retinol (Vitamin A)	Cofactors and Vitamins	Vitamin A Metabolism	-0.1667	0.02018	-2.3425	0.18608
1-palmitoyl-GPE (16:0)	Lipid	Lysophospholipid	-0.1696	0.01806	-2.3849	0.17461
glutamine	Amino Acid	Glutamate Metabolism	-0.1701	0.01774	-2.3915	0.17398
xylose	Carbohydrate	Pentose Metabolism	-0.1711	0.01709	-2.4058	0.17165
gamma-glutamylglycine	Peptide	Gamma-glutamyl Amino Acid	-0.172	0.0165	-2.4189	0.16829
1-oleoyl-GPE (18:1)	Lipid	Lysophospholipid	-0.172	0.01646	-2.4197	0.16829
sphingomyelin (d17:1/14:0, d16:1/15:0)*	Lipid	Sphingomyelins	-0.1728	0.01598	-2.431	0.16681

BIOCHEMICAL	Amino Acids	Sub Pathway	Estimate	*p*-value	Statistics	FDR
sarcosine	Amino Acid	Glycine, Serine and Threonine Metabolism	-0.1738	0.01538	-2.4452	0.16445
1-oleoyl-2-docosahexaenoyl-GPE (18:1/22:6)*	Lipid	Phosphatidylethanolamine (PE)	-0.1751	0.01463	-2.4638	0.16031
beta-sitosterol	Lipid	Sterol	-0.1767	0.01371	-2.4876	0.15411
gamma-glutamylserine	Peptide	Gamma-glutamyl Amino Acid	-0.1789	0.01255	-2.5199	0.1463
glycosyl-N-tricosanoyl-sphingadienine (d18:2/23:0)*	Lipid	Hexosylceramides (HCER)	-0.1797	0.01218	-2.531	0.14623
methionine sulfoxide	Amino Acid	Methionine, Cysteine, SAM and Taurine Metabolism	-0.1808	0.01165	-2.5471	0.14415
1-palmitoleoyl-2-linolenoyl-GPC (16:1/18:3)*	Lipid	Phosphatidylcholine (PC)	-0.1818	0.0112	-2.5613	0.1438
heneicosapentaenoate (21:5n3)	Lipid	Long Chain Polyunsaturated Fatty Acid (n3 and n6)	-0.1827	0.01078	-2.5748	0.1438
1-linoleoyl-2-arachidonoyl-GPC (18:2/20:4n6)*	Lipid	Phosphatidylcholine (PC)	-0.1831	0.01062	-2.5801	0.1438
S-1-pyrroline-5-carboxylate	Amino Acid	Glutamate Metabolism	-0.1832	0.01057	-2.5818	0.1438
palmitoyl dihydrosphingomyelin (d18:0/16:0)*	Lipid	Dihydrosphingomyelins	-0.1838	0.01033	-2.5902	0.1438
threonine	Amino Acid	Glycine, Serine and Threonine Metabolism	-0.184	0.01021	-2.5945	0.1438
lactosyl-N-behenoyl-sphingosine (d18:1/22:0)*	Lipid	Lactosylceramides (LCER)	-0.1847	0.00992	-2.6045	0.1438
campesterol	Lipid	Sterol	-0.1875	0.00885	-2.6447	0.13976

BIOCHEMICAL	Amino Acids	Sub Pathway	Estimate	*p*-value	Statistics	FDR
1-(1-enyl-palmitoyl)-GPC (P-16:0)*	Lipid	Lysoplasmalogen	-0.1897	0.00806	-2.6774	0.13037
2-methylserine	Amino Acid	Glycine, Serine and Threonine Metabolism	-0.1917	0.00742	-2.706	0.1246
dopamine 3-O-sulfate	Amino Acid	Tyrosine Metabolism	-0.1926	0.00712	-2.7202	0.12138
N-oleoylserine	Lipid	Endocannabinoid	-0.1938	0.00677	-2.7377	0.11809
2'-O-methylcytidine	Nucleotide	Pyrimidine Metabolism, Cytidine containing	-0.1943	0.00665	-2.7438	0.11754
glycosyl ceramide (d18:1/20:0, d16:1/22:0)*	Lipid	Hexosylceramides (HCER)	-0.1948	0.00648	-2.7524	0.11754
docosahexaenoylcarnitine (C22:6)*	Lipid	Fatty Acid Metabolism (Acyl Carnitine, Polyunsaturated)	-0.1952	0.00638	-2.7578	0.11754
myristoyl dihydrosphingomyelin (d18:0/14:0)*	Lipid	Dihydrosphingomyelins	-0.2002	0.00513	-2.8312	0.10577
hydroxy-CMPF*	Lipid	Fatty Acid, Dicarboxylate	-0.2018	0.00477	-2.8552	0.10551
isovalerylglycine	Amino Acid	Leucine, Isoleucine and Valine Metabolism	-0.2021	0.00471	-2.8598	0.10551
S-methylcysteine sulfoxide	Amino Acid	Methionine, Cysteine, SAM and Taurine Metabolism	-0.2138	0.00276	-3.033	0.07614
glycerate	Carbohydrate	Glycolysis, Gluconeogenesis, and Pyruvate Metabolism	-0.2151	0.0026	-3.0517	0.07329
carotene diol (2)	Cofactors and Vitamins	Vitamin A Metabolism	-0.2177	0.0023	-3.0903	0.06768

BIOCHEMICAL	Amino Acids	Sub Pathway	Estimate	*p*-value	Statistics	FDR
asparagine	Amino Acid	Alanine and Aspartate Metabolism	-0.2222	0.00185	-3.1573	0.06086
carotene diol (1)	Cofactors and Vitamins	Vitamin A Metabolism	-0.2275	0.00142	-3.2379	0.05375
1-oleoyl-GPC (18:1)	Lipid	Lysophospholipid	-0.229	0.00132	-3.2595	0.05285
histidine	Amino Acid	Histidine Metabolism	-0.2307	0.00121	-3.2846	0.05085
1-methylnicotinamide	Cofactors and Vitamins	Nicotinate and Nicotinamide Metabolism	-0.2379	0.00084	-3.3944	0.04069
propionylglycine (C3)	Lipid	Fatty Acid Metabolism (also BCAA Metabolism)	-0.2391	0.00079	-3.4118	0.04014
serine	Amino Acid	Glycine, Serine and Threonine Metabolism	-0.2451	0.00057	-3.5025	0.03167
1-palmitoyl-2-docosahexaenoyl-GPC (16:0/22:6)	Lipid	Phosphatidylcholine (PC)	-0.2461	0.00054	-3.5177	0.03132
glycine	Amino Acid	Glycine, Serine and Threonine Metabolism	-0.2496	0.00045	-3.5709	0.02839
1-(1-enyl-palmitoyl)-2-oleoyl-GPC (P-16:0/18:1)*	Lipid	Plasmalogen	-0.2573	0.00029	-3.6899	0.01937
1-oleoyl-2-docosahexaenoyl-GPC (18:1/22:6)*	Lipid	Phosphatidylcholine (PC)	-0.2821	6.73E-05	-4.075	0.00744
N-acetylglycine	Amino Acid	Glycine, Serine and Threonine Metabolism	-0.296	2.79E-05	-4.2937	0.00423
threonate	Cofactors and Vitamins	Ascorbate and Aldarate Metabolism	-0.3113	9.92E-06	-4.5397	0.00234
oxalate (ethanedioate)	Cofactors and Vitamins	Ascorbate and Aldarate Metabolism	-0.3146	7.93E-06	-4.592	0.00234

BIOCHEMICAL	Amino Acids	Sub Pathway	Estimate	*p*-value	Statistics	FDR
phosphocholine	Lipid	Phospholipid Metabolism	-0.3158	7.29E-06	-4.6113	0.00234
